# Supplementary figures and images for: Unraveling radiation resistance strategies in two bacterial strains from the high background radiation area of Chavara-Neendakara: A comprehensive whole genome analysis
Source: PLoS One. 2024 Jun 10;19(6):e0304810. doi: 10.1371/journal.pone.0304810 (PMC11164402; doi:10.1371/journal.pone.0304810)

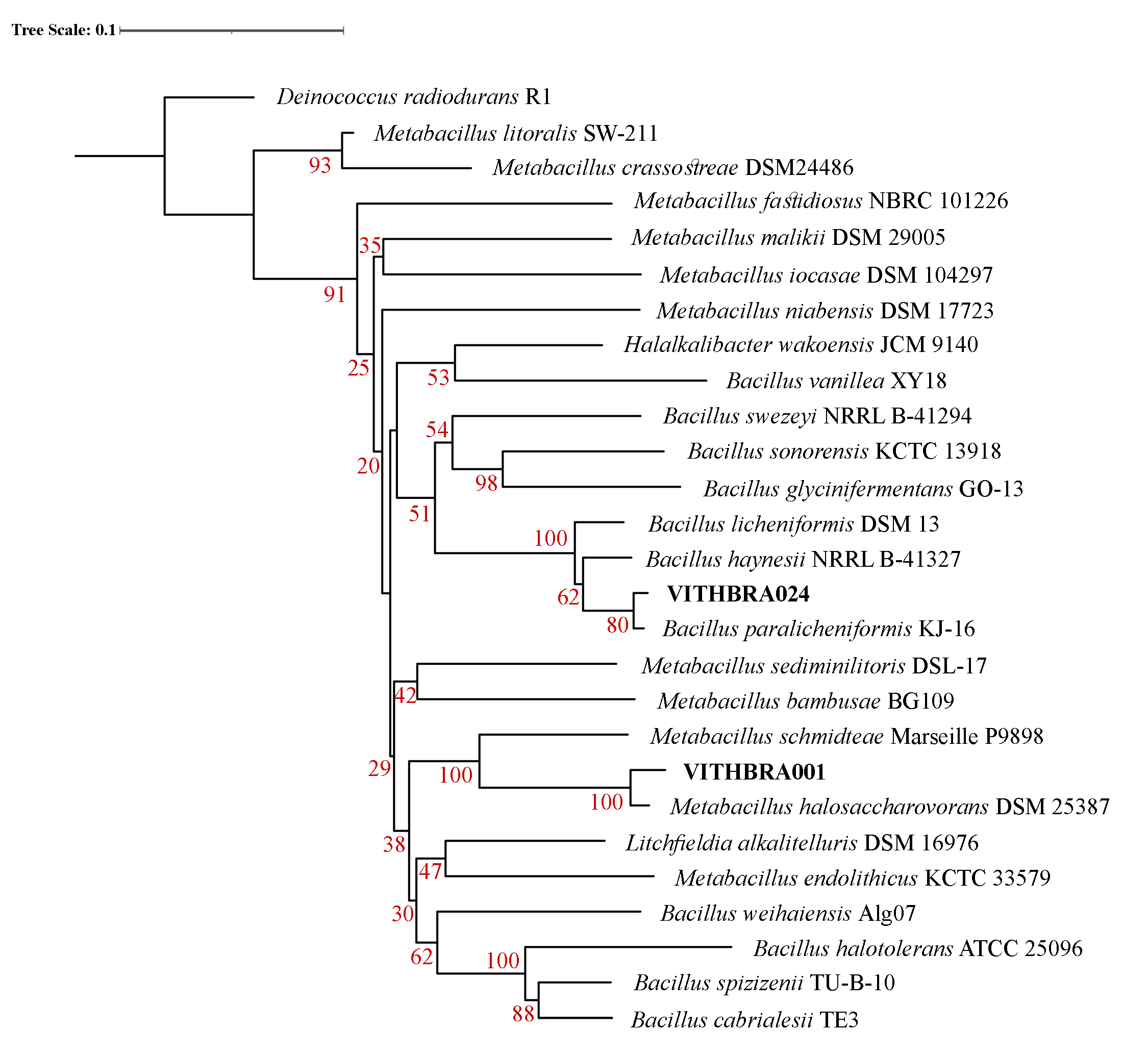

Supplement: S1 Fig — The tree was built using TYGS platform using the closely allied type species of each strain (observed in Fig 2) and D. radiodurans as outgroup. (TIF) [file pone.0304810.s001.tif]

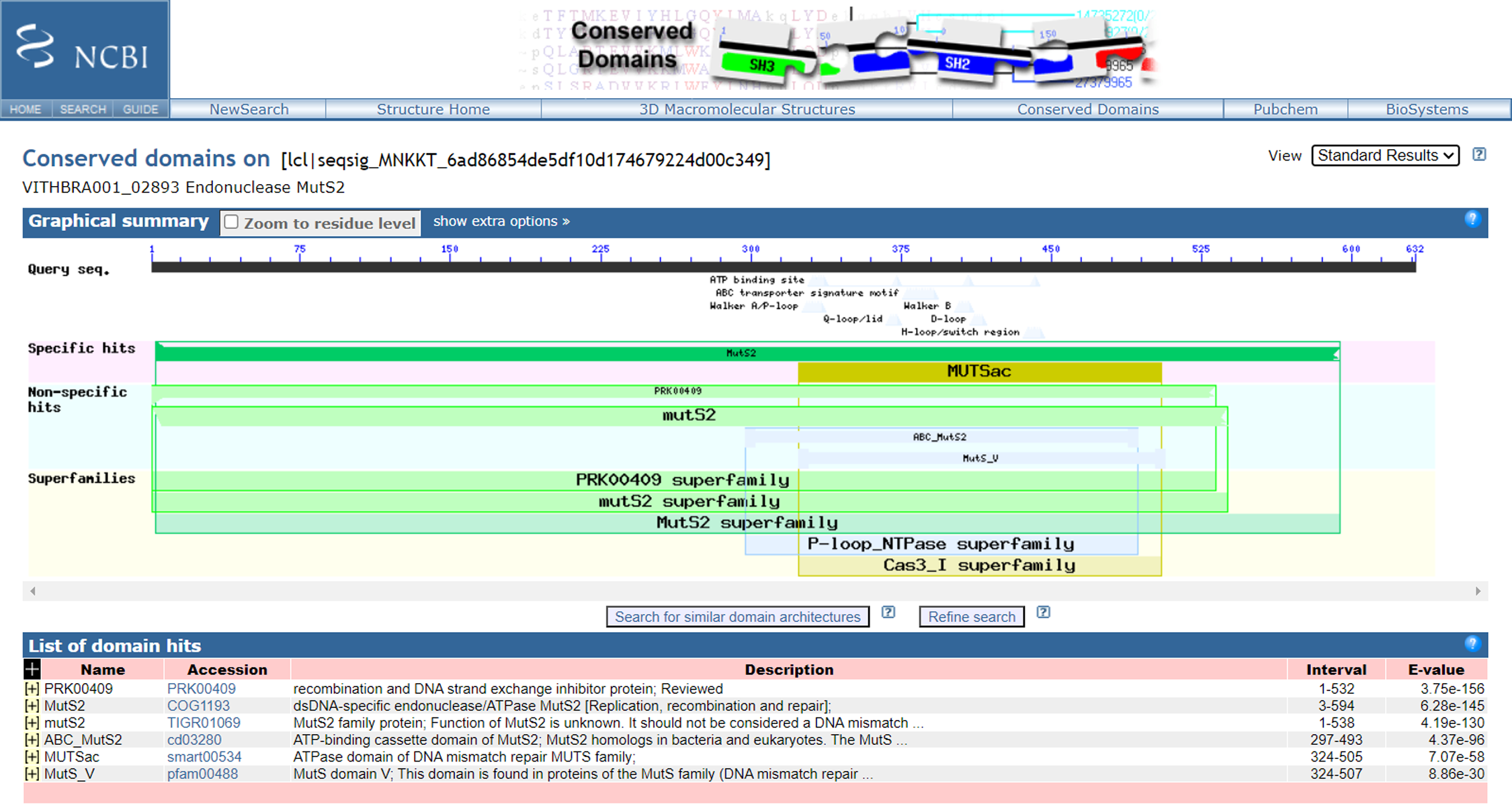

Supplement: S2 Fig — Identifying the protein VITHBRA001_02893 having MutS2 domain but the C-terminal region does not have the small MutS2 region (SMR) like the other homolog VITHBRA001_01182. (TIF) [file pone.0304810.s002.tif]

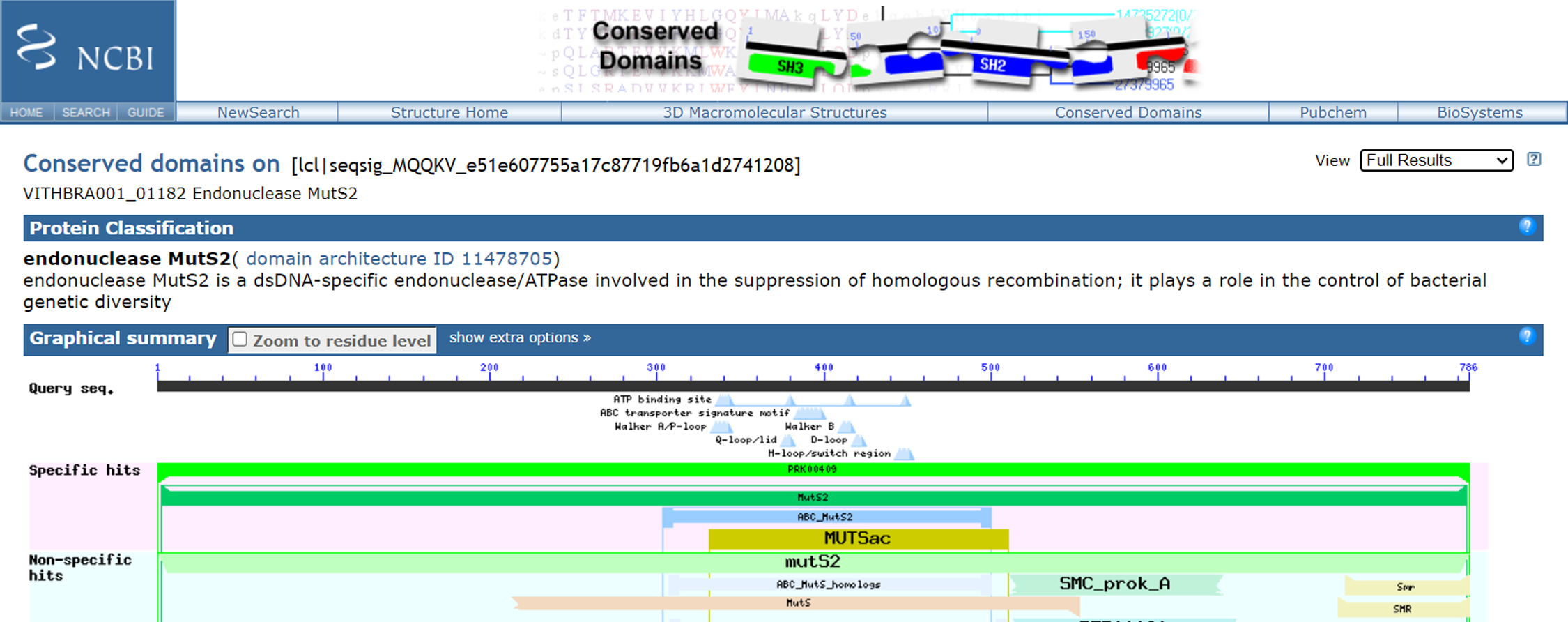

Supplement: S3 Fig — VITHBRA001_01182 have the SMR region which can be seen in C-terminal region in this figure. (TIF) [file pone.0304810.s003.tif]

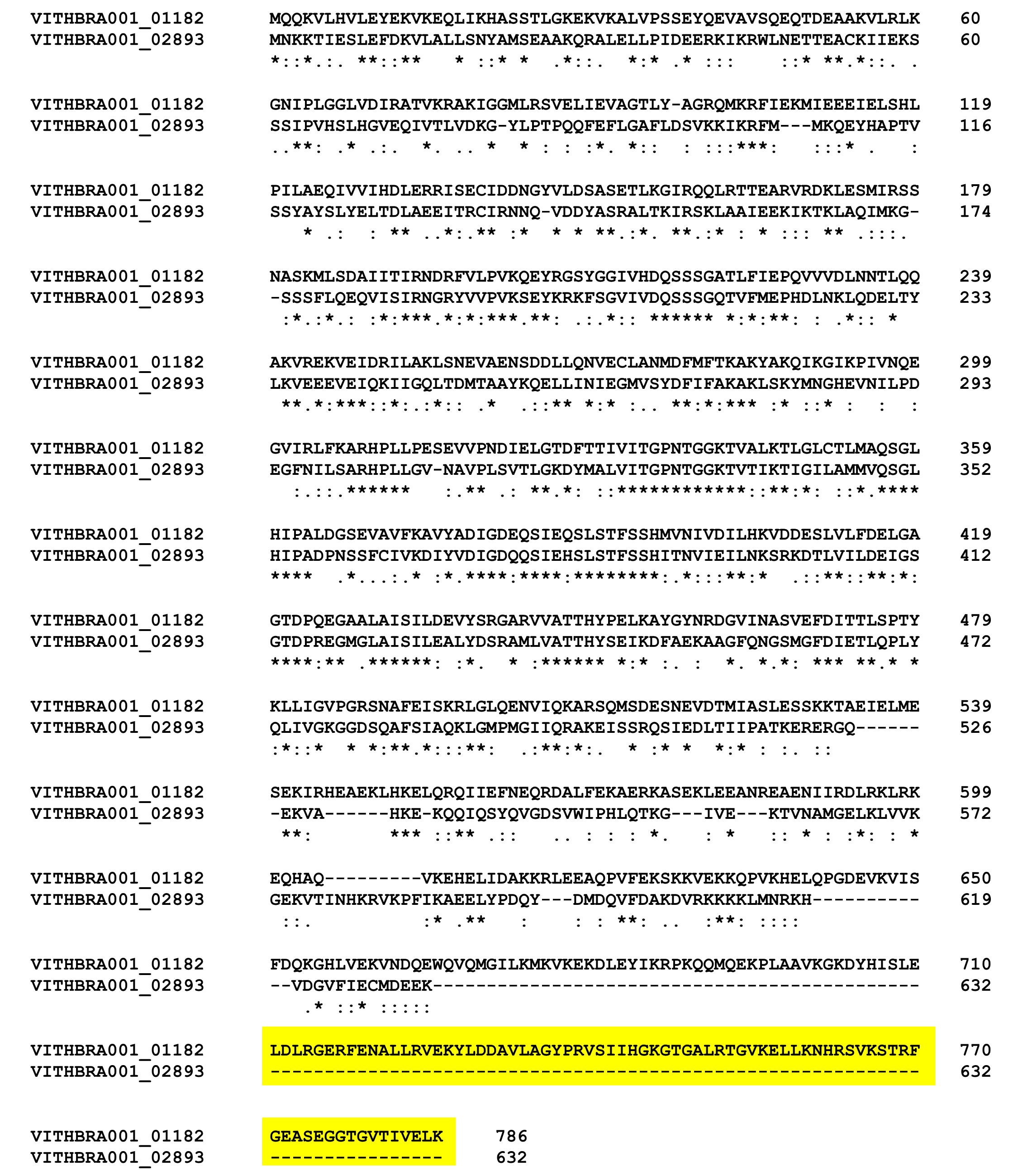

Supplement: S4 Fig — The highlighted section (in yellow) shows the absence of small MutS2 region (SMR) in VITHBRA001_02893 as compared to VITHBRA001_01182. The MSA was performed using Clustal omega version 1.2.2. (TIF) [file pone.0304810.s004.tif]

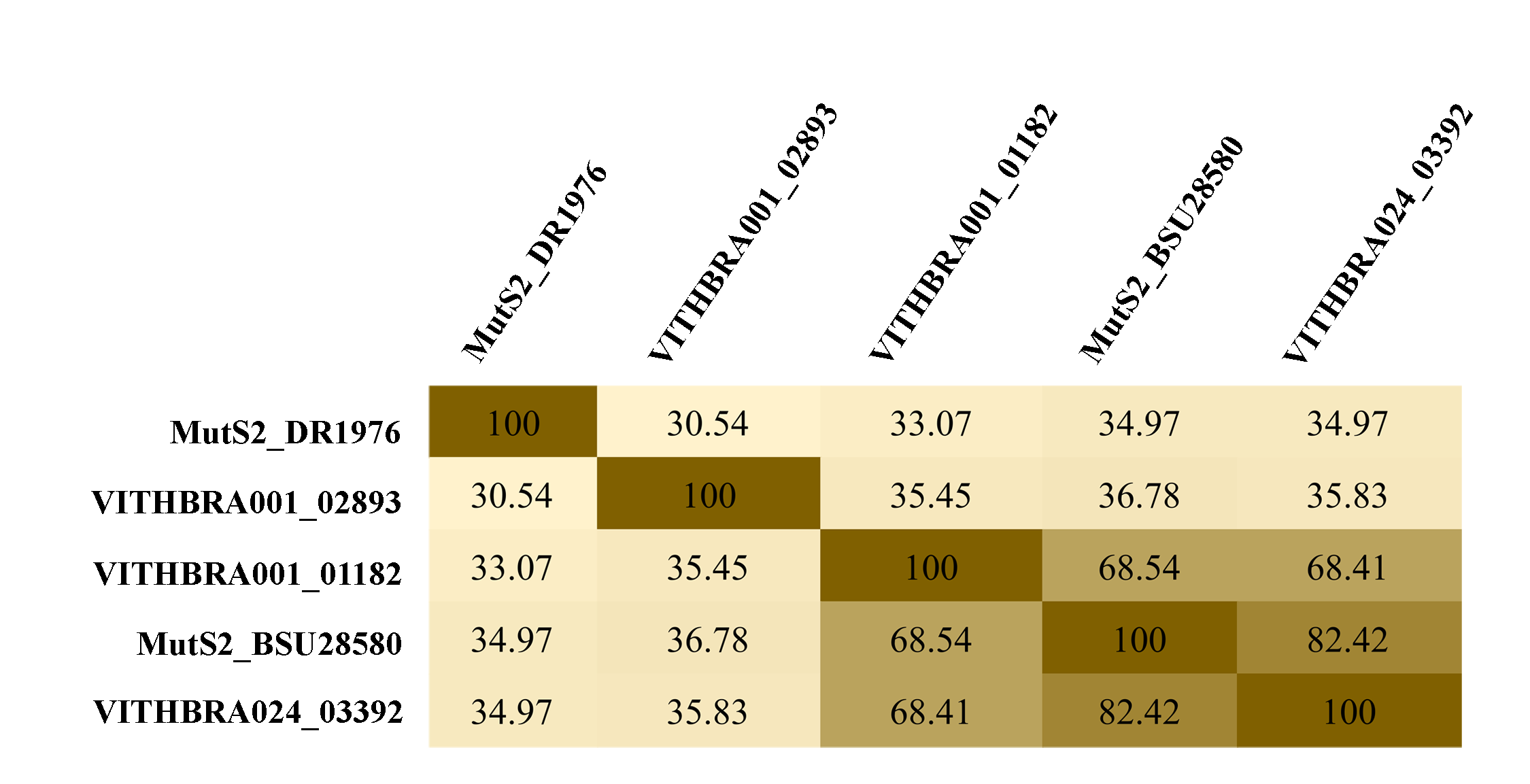

Supplement: S5 Fig — The MutS2 proteins for both the strains have been compared with MutS2 of D. radiodurans and B. subtilis to hypothecate the function of MutS2 of candidate strains. The aforementioned two species is reported to have different function of MutS2. The percentage identity suggests that the strains in this study have more identity with B. subtilis MutS2 as compared to D. radiodurans. Hence, the function of MutS2 of strains in this study could be similar to the function of B. subtilis MutS2. The deeper the color of the matrix the more the identity. The percentage identity was calculated using Uniprot align tool. (TIF) [file pone.0304810.s005.tif]

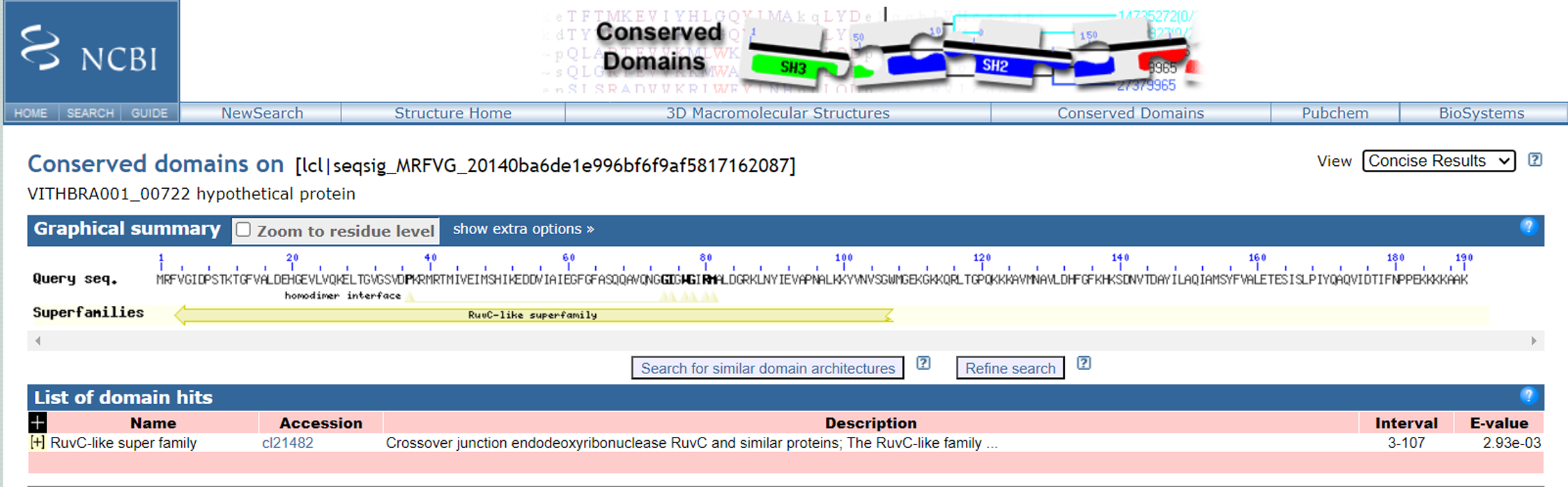

Supplement: S6 Fig — It is identified as protein (VITHBRA001_00722) belonging from the RuvC-like superfamily. This could be a part of RuvABC system that helps in resolution of Holliday junctions formed in homologous recombination repair of DNA. (TIF) [file pone.0304810.s006.tif]

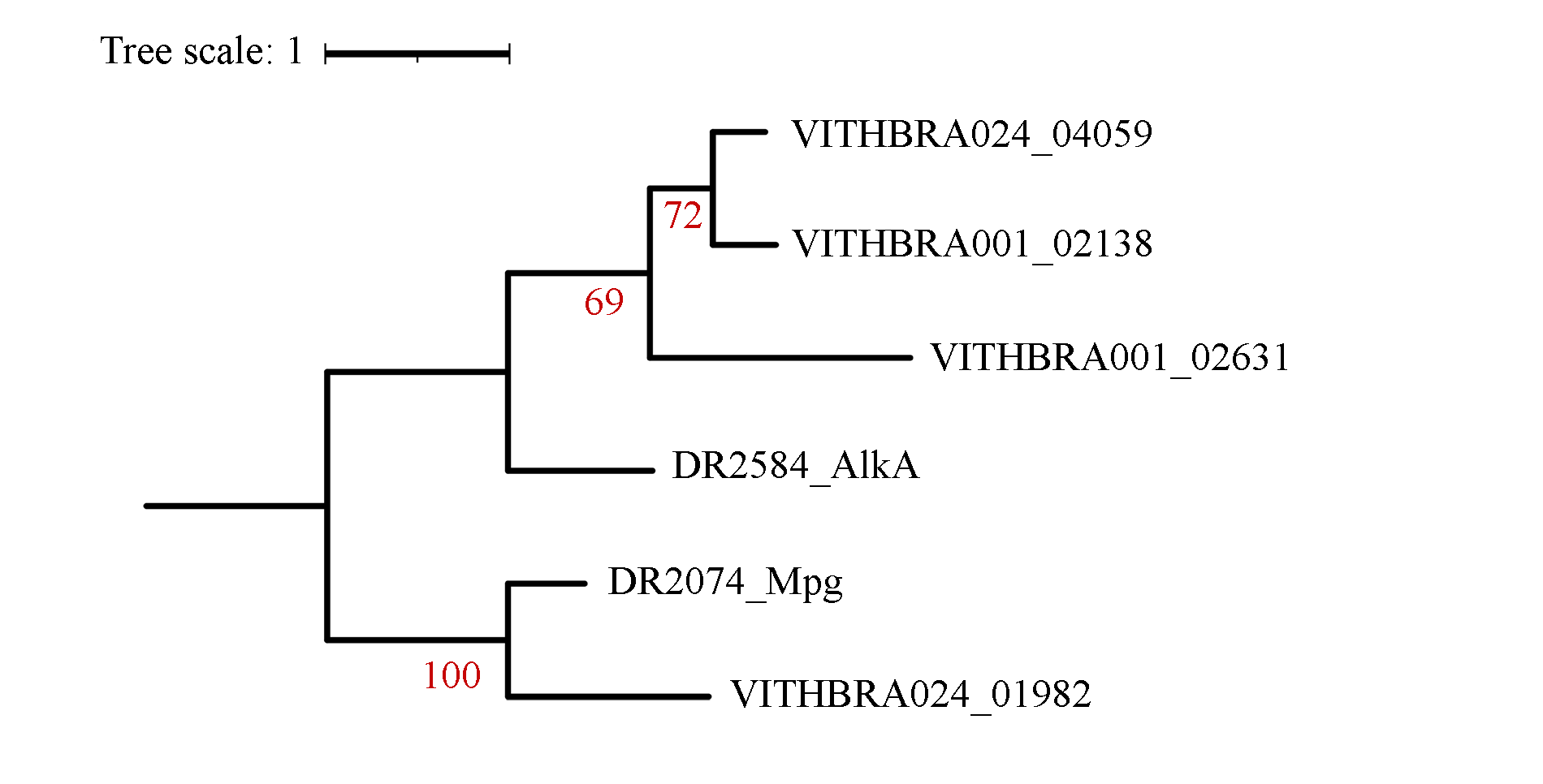

Supplement: S7 Fig — Through this maximum likelihood phylogenetic tree, we could conclude that two proteins of VITHBRA001 belong to 3-methyladenine DNA glycosylase II (AlkA) whereas VITHBRA024 have one protein with 3-methyladenine DNA glycosylase I (Mpg) category and other with 3-methyladenine DNA glycosylase II (AlkA) category. The D. radiodurans’s proteins were used to identify the protein types. The tree was constructed using MEGA X with 1000 bootstrap support. (TIF) [file pone.0304810.s007.tif]

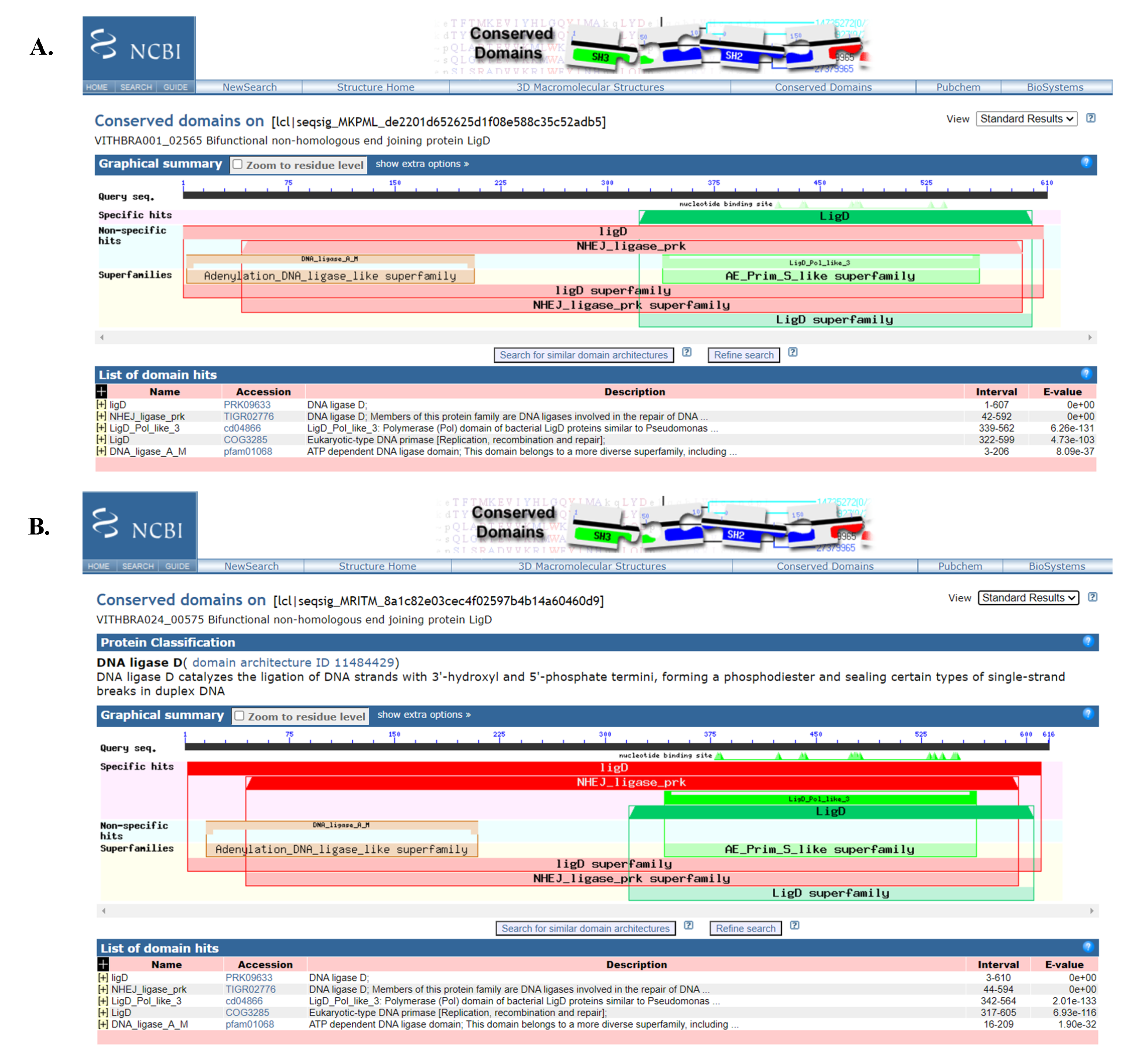

Supplement: S8 Fig — Figures A and B belong to ligase LigD of VITHBRA001 and VITHBRA024 respectively. In both the cases we see that the proteins show presence of two function: the N-terminal ligase function and the C-terminal polymerase function. (TIF) [file pone.0304810.s008.tif]

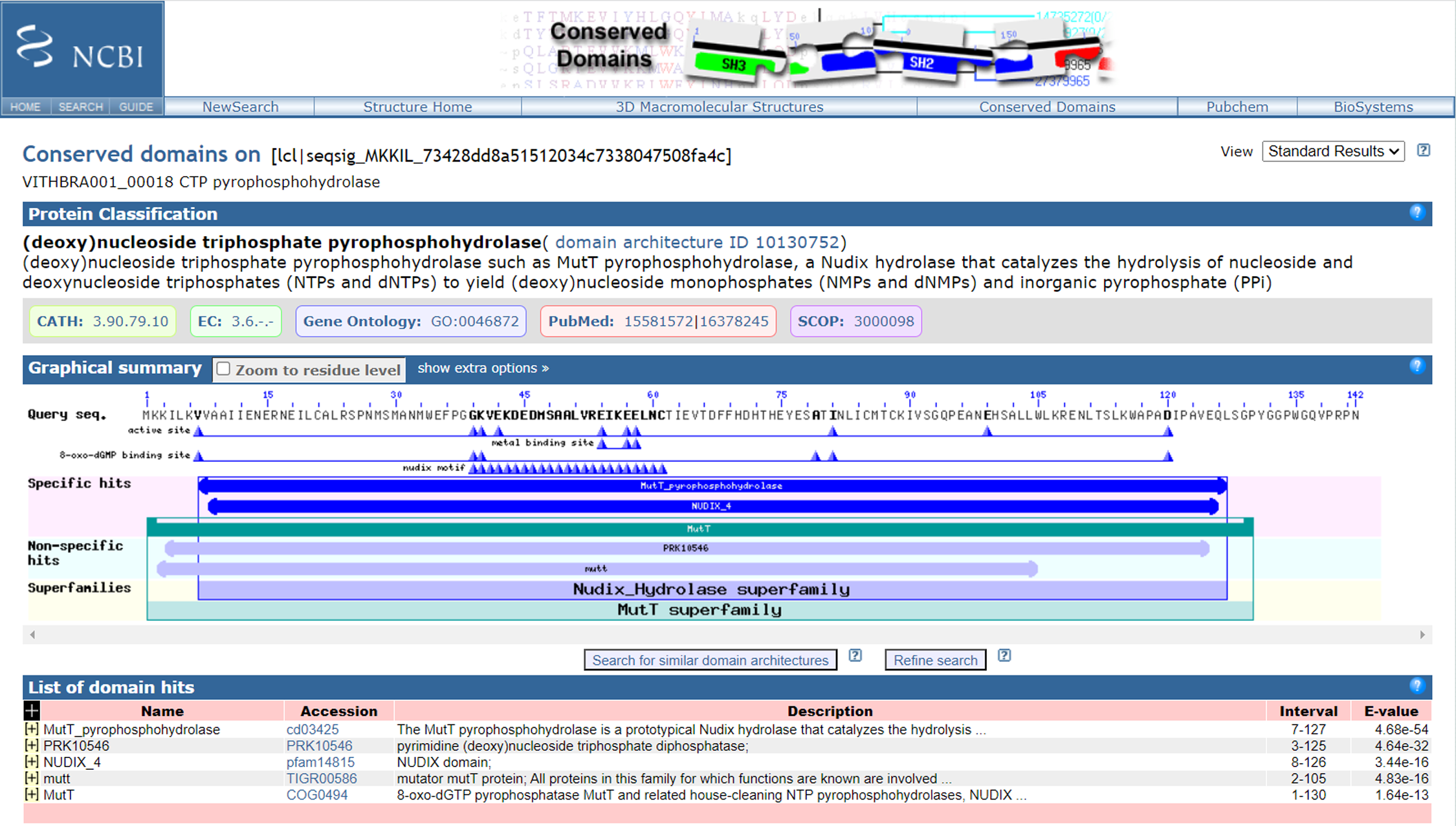

Supplement: S9 Fig — The analysis identifies the protein VITHBRA001_00018 as a MutT prototypical Nudix hydrolase protein which removes the oxidized guanine product 8-oxoG form the free nucleotide pool. (TIF) [file pone.0304810.s009.tif]

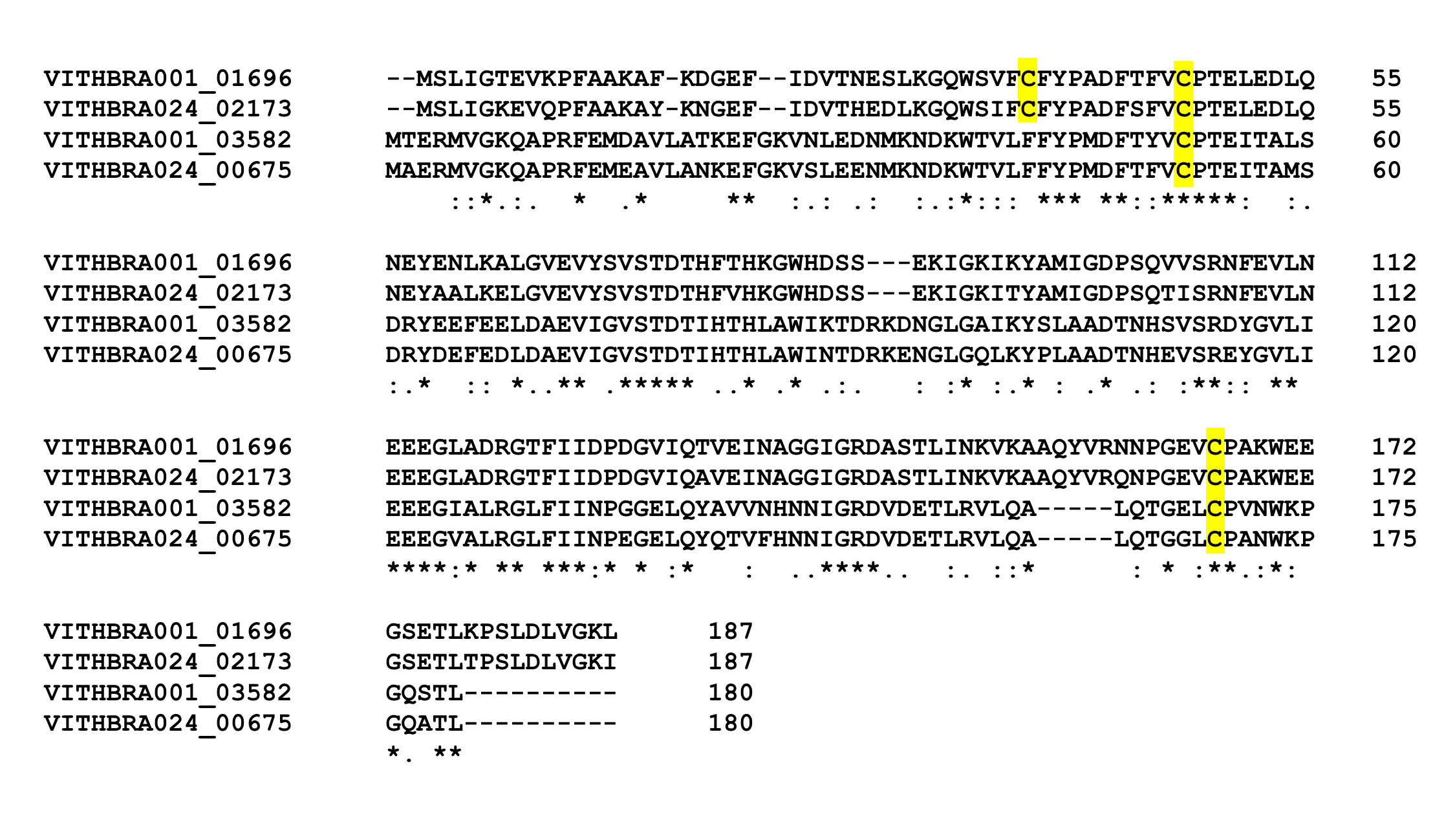

Supplement: S10 Fig — The first two sequences belong to atypical 3-cysteine containing AhpC and the last two belong to the typical 2-cysteine containing AhpC. The highlights (in yellow) show the presence of cysteine in the two types of AhpC. The MSA was performed using Clustal omega version 1.2.2. (TIF) [file pone.0304810.s010.tif]

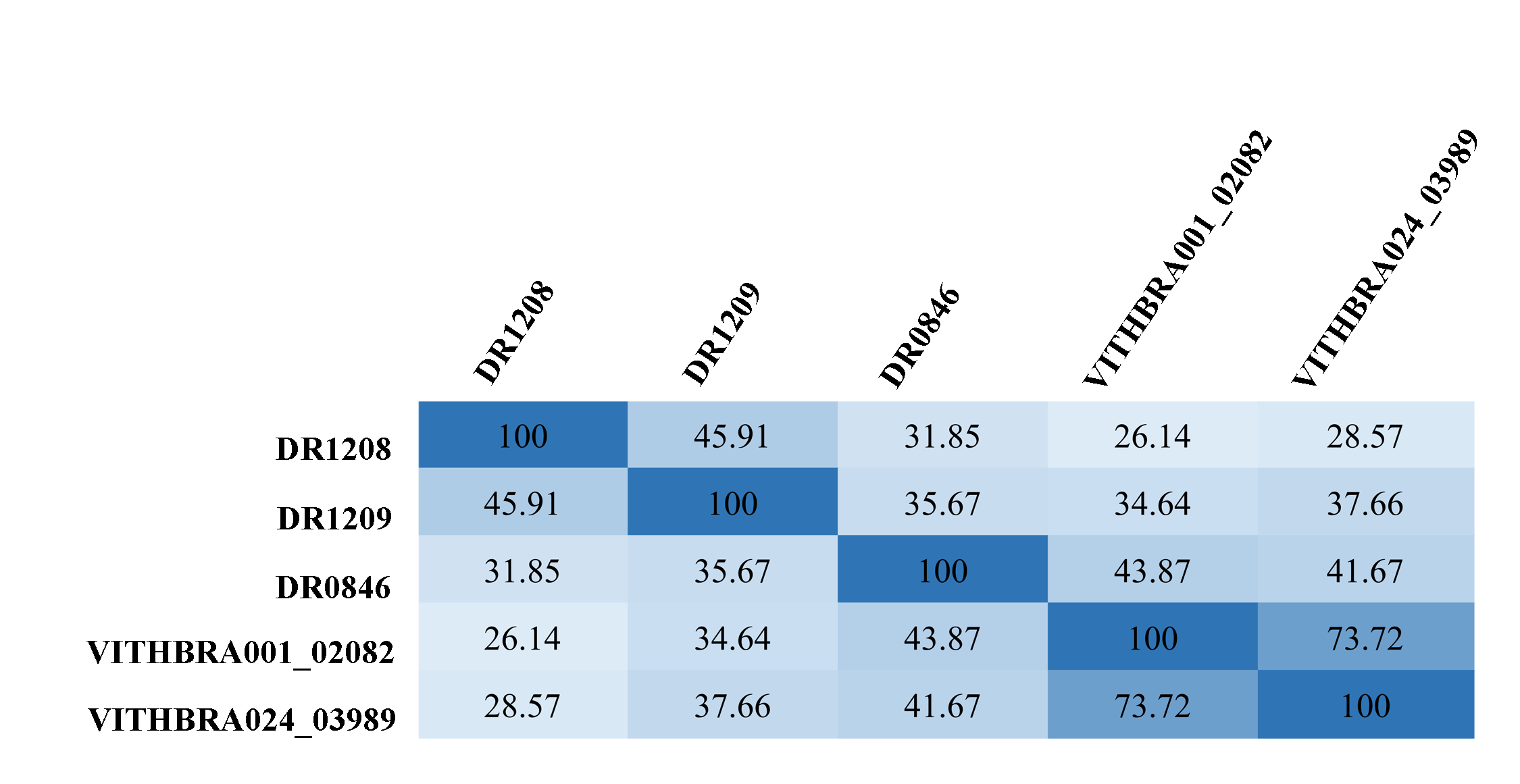

Supplement: S11 Fig — The three Bcp homologs in D. radiodurans are compared with the one Bcp protein identified in each VITHBRA001 and VITHBRA024. It was observed that the Bcp of the candidate strains have more identity with the D. radiodurans’s DR0846 Bcp protein. The deeper the colour in the matrix the more is the identity of the proteins. The percentage identity was calculated using Uniprot align tool. (TIF) [file pone.0304810.s011.tif]

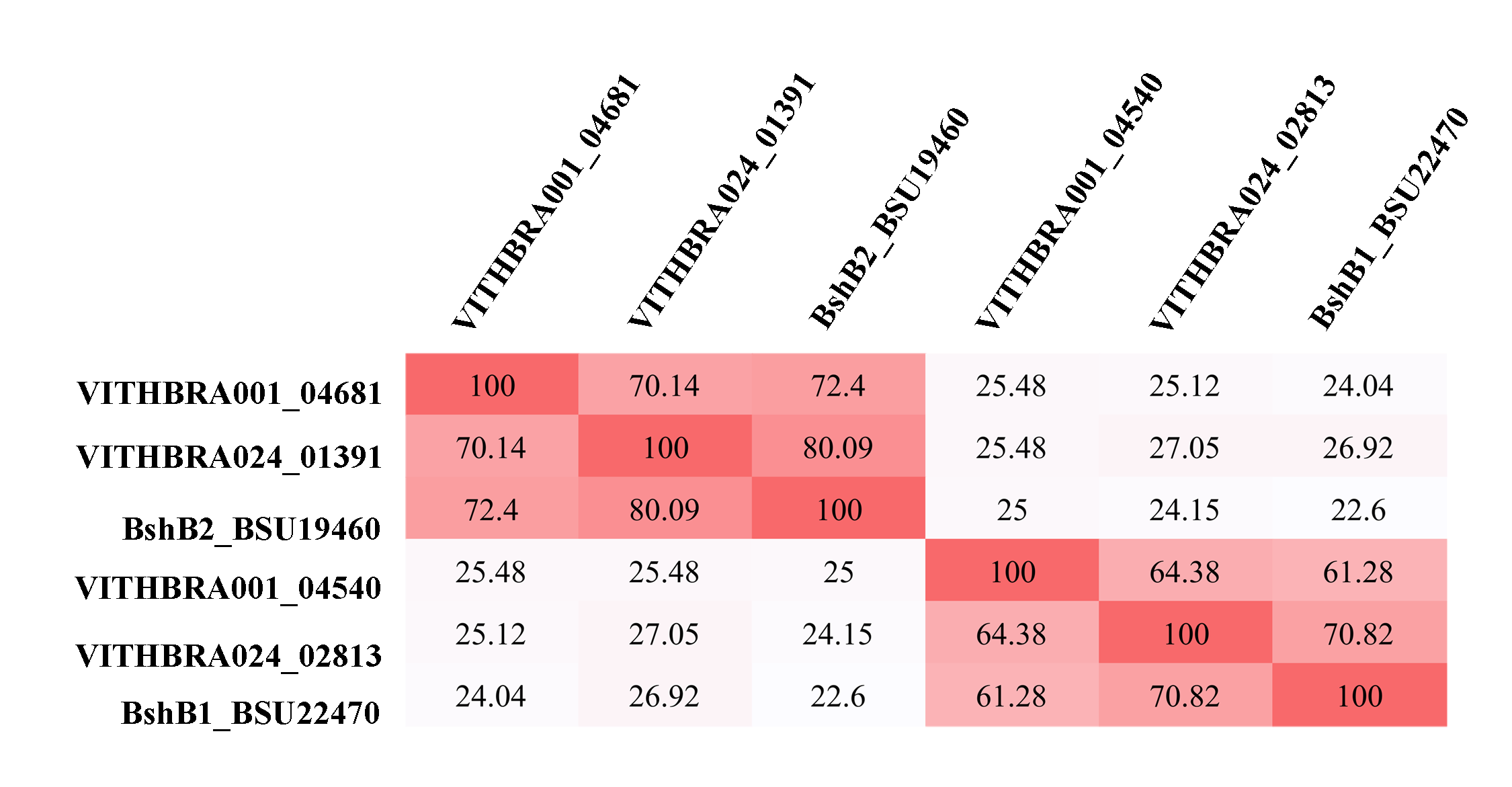

Supplement: S12 Fig — The matrix helped segregate the annotation of the two homologs of BshB genes present in the candidate strains with the help of BshB homologs of B. subtilis which has already been studied and reported. The deeper the colour in the matrix the more is the identity of the proteins. The percentage identity was calculated using Uniprot align tool. (TIF) [file pone.0304810.s012.tif]

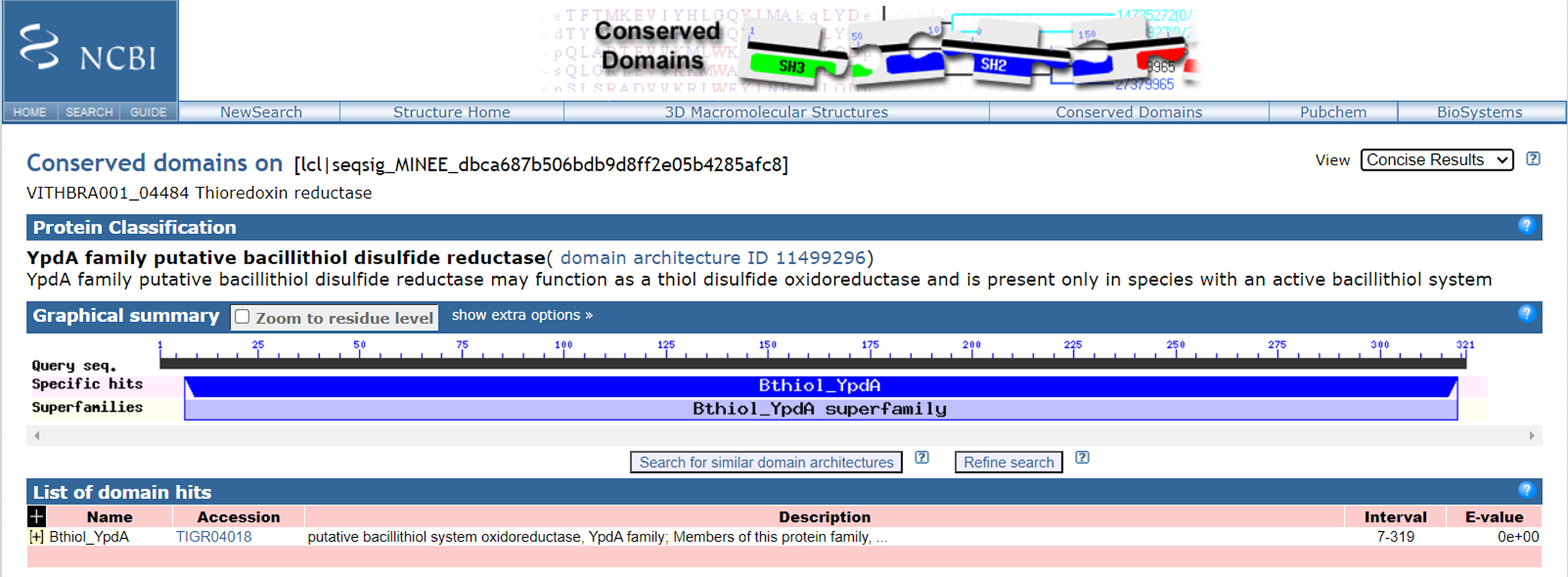

Supplement: S13 Fig — The sequence VITHBRA001_04484 is annotated as a homolog of thioredoxin reductase (TrxB) by Prokka which is identified and confirmed by NCBI CD search as Bacillithiol disulphide reductase (YpdA). (TIF) [file pone.0304810.s013.tif]

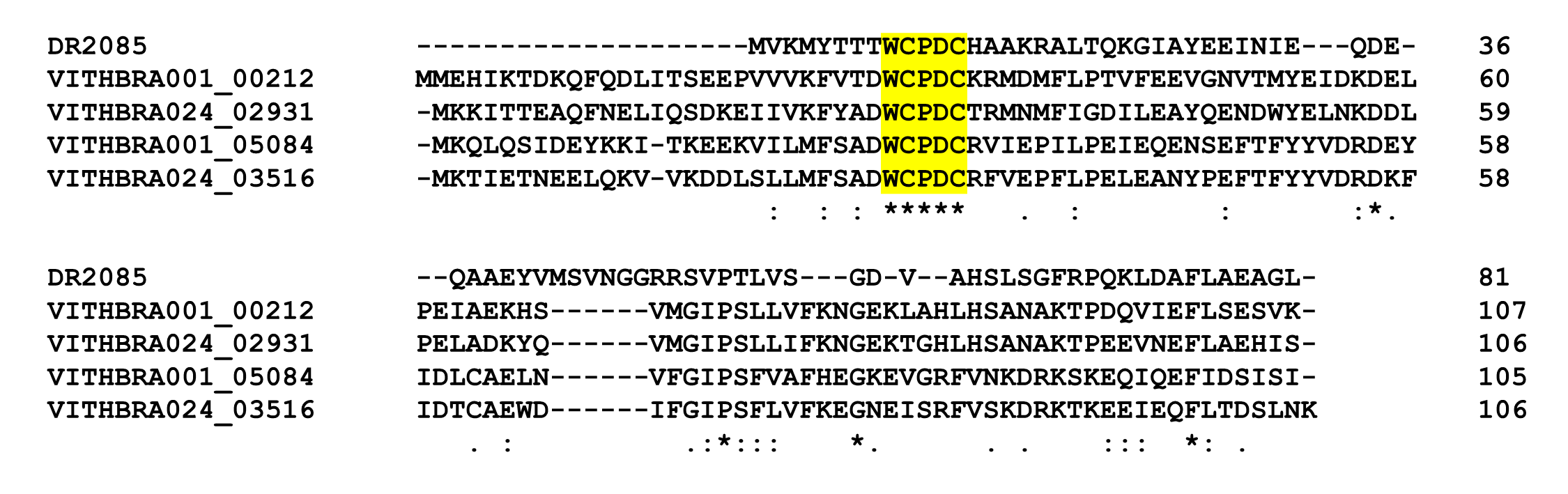

Supplement: S14 Fig — Multiple sequence alignment shows the WCPDC motif (highlighted in yellow) present in the two trx-like genes of both the strains which is also the motif present in a Trx-like protein of D. radiodurans which is reported to be expressed after radiation stress. (TIF) [file pone.0304810.s014.tif]

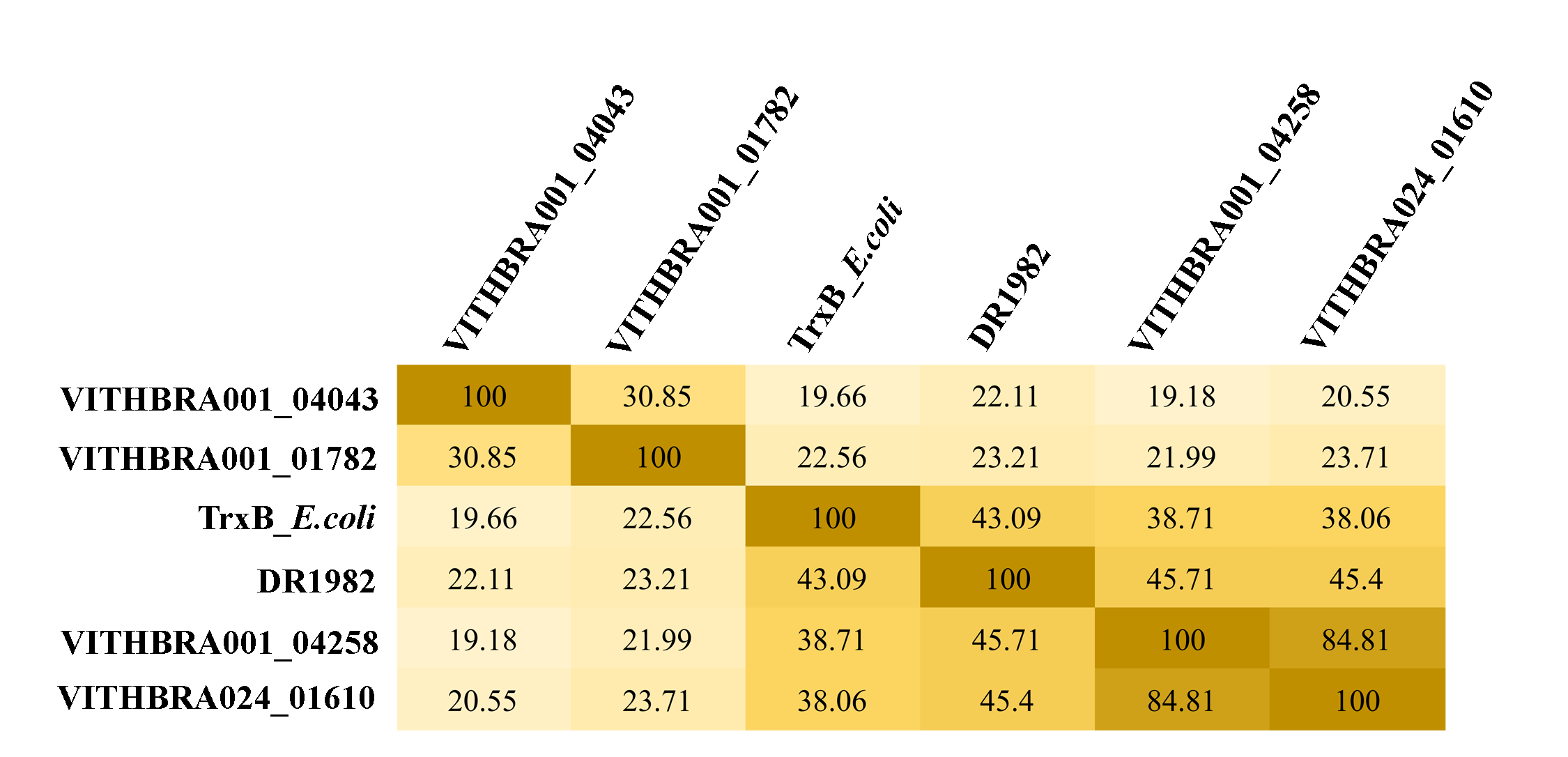

Supplement: S15 Fig — Three homologs of TrxB genes are present in VITHBRA001 and a comparison with D. radiodurans and E. coli TrxR gene helped to identify which homolog has more identity with TrxR of D. radiodurans. The deeper the colour in the matrix the more is the identity of the proteins. The percentage identity was calculated using Uniprot align tool. (TIF) [file pone.0304810.s015.tif]

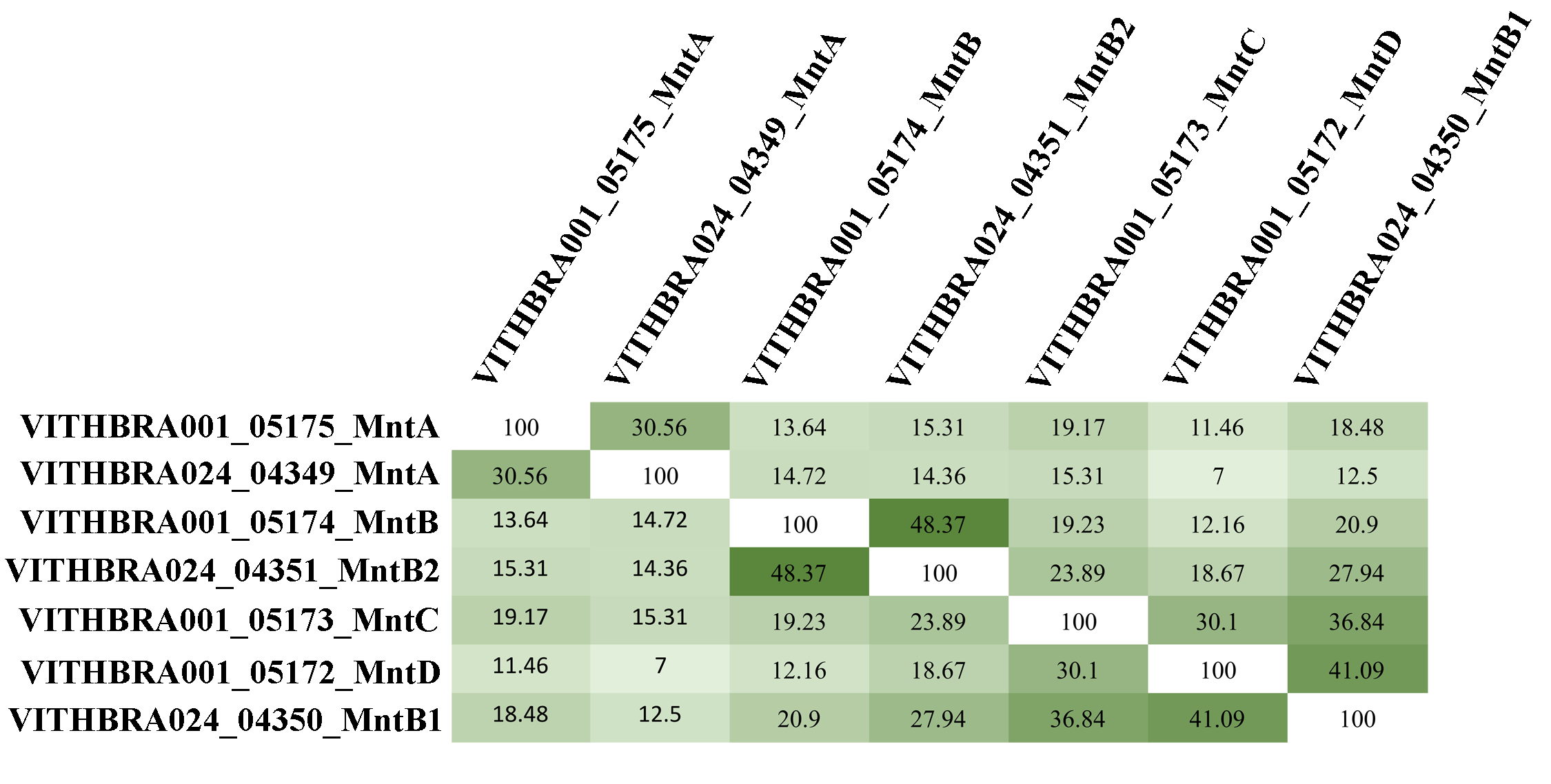

Supplement: S16 Fig — Through percentage identity it was observed that MntB1 had more identity to MntD of VITHBRA001 than to MntC. However, both the proteins (MntC and MntD) seem to have the permease activity in the MntABC complex hence, MntB1 would have permease activity. The deeper the colour in the matrix the more is the identity of the proteins. The percentage identity was calculated using Uniprot align tool. (TIF) [file pone.0304810.s016.tif]
