# Supplementary material for: Unraveling radiation resistance strategies in two bacterial strains from the high background radiation area of Chavara-Neendakara: A comprehensive whole genome analysis
Source: PLoS One. 2024 Jun 10;19(6):e0304810. doi: 10.1371/journal.pone.0304810 (PMC11164402; doi:10.1371/journal.pone.0304810)
Supplement: S1 Table — (DOCX) [file pone.0304810.s018.docx]

**S1 Table. Standalone BLAST results between C_30_ carotenoid biosynthesis proteins of various bacteria and protein FASTA file of VITHBRA001.**

| **VITHBRA001 locus ID** | **Annotation of VITHBRA001 proteins** | **Match with other organisms** | **Percentage identity** | **E-value** |
| --- | --- | --- | --- | --- |
| Prokka-VITHBRA001_02657  NCBI- MCV9886562.1 | Prokka- 4,4’-diapophytoene desaturase  NCBI- phytoene desaturase family protein | *H. halophilus* CrtNa | 34.42 | 3.76e-97 |
|  |  | *H. halophilus* CrtNb | 29.84 | 2.12e-76 |
|  |  | *H. halophilus* CrtNc | 28.37 | 8.67e-72 |
|  |  | ***S. aureus* CrtN** | **45.78** | **7.44e-144** |
|  |  | *M. indicus* CrtNa | 33.13 | 1.04e-95 |
|  |  | *M. indicus* CrtNb | 30.95 | 5.07e-75 |
|  |  | *M. indicus* CrtNc | 32.6 | 1.97e-92 |
|  |  | *M. flavus* CrtNa | 34.41 | 2.01e-100 |
|  |  | *M. flavus* CrtNb | 31.39 | 1.07e-81 |
|  |  | *M. flavus* CrtNc | 31.52 | 1.91e-88 |
| Prokka-VITHBRA001_02656  NCBI- MCV9886561.1 | Prokka- 4,4’-diapophytoene synthase  NCBI- phytoene/squalene synthase family protein | ***H. halophilus* CrtM** | **43.35** | **1.13e-72** |
|  |  | *S. aureus* CrtM | 35.16 | 2.53e-46 |
|  |  | *M. indicus* CrtM | 41.33 | 6.61e-69 |
|  |  | ***M. flavus* CrtM** | **42.86** | **1.31e-71** |
| Prokka-VITHBRA001_02655  NCBI- MCV9886560.1 | Prokka- 4,4’-diaponeurosporene oxygenase  NCBI- phytoene desaturase family protein | *H. halophilus* CrtNa | 28.14 | 1.06e-63 |
|  |  | *H. halophilus* CrtNb | 32.52 | 5.59e-79 |
|  |  | *H. halophilus* CrtNc | 25.108 | 7.38e-45 |
|  |  | *S. aureus* CrtN | 26.92 | 2.59e-55 |
|  |  | ***S. aureus* CrtP** | **59.39** | **0.0** |
|  |  | *M. indicus* CrtNa | 27.24 | 1.43e-58 |
|  |  | *M. indicus* CrtNb | 35.23 | 1.43e-92 |
|  |  | *M. indicus* CrtNc | 25.66 | 3.8e-48 |
|  |  | *M. flavus* CrtNa | 28.11 | 2.45e-59 |
|  |  | *M. flavus* CrtNb | 33.198 | 1.03e-87 |
|  |  | *M. flavus* CrtNc | 24.52 | 3.66e-47 |
| Prokka-VITHBRA001_02654  NCBI- MCV9886559.1 | Prokka- 4,4’-diaponeurosporenoate glycosyltransferase  NCBI- glycosyltransferase family 2 protein | *H. halophilus* orf-GT | 24.202 | 2.07e-23 |
|  |  | ***S. aureus* CrtQ** | **37.78** | **6.53e-74** |
| Prokka-VITHBRA001_02653  NCBI- MCV9886558.1 | Prokka- Hypothetical protein  NCBI- glycosyl-4,4’- diaponeurosporenoate acyltransferase | ***S. aureus* CrtO** | **36.62** | **1.21e-29** |
| Prokka-VITHBRA001_03534  NCBI- MCV9887402.1 | Prokka- 4,4’-diaponeurosporene oxygenase  NCBI- phytoene desaturase family protein | *H. halophilus* CrtNa | 29.33 | 2.66e-66 |
|  |  | *H. halophilus* CrtNb | 32.11 | 2.31e-82 |
|  |  | *H. halophilus* CrtNc | 27.39 | 3.84e-48 |
|  |  | *S. aureus* CrtN | 28.63 | 1.64e-70 |
|  |  | ***S. aureus* CrtP** | **43.26** | **6.49e-140** |
|  |  | *M. indicus* CrtNa | 29.209 | 4.14e-68 |
|  |  | *M. indicus* CrtNb | 35.44 | 1.95e-99 |
|  |  | *M. indicus* CrtNc | 28.016 | 1.33e-52 |
|  |  | *M. flavus* CrtNa | 29.006 | 7.75e-71 |
|  |  | *M. flavus* CrtNb | 34.69 | 1.87e-97 |
|  |  | *M. flavus* CrtNc | 26.907 | 4.63e-53 |

The proteins with highest identity and lowest e-values are mentioned in bold.
