# Supplementary material for: Unraveling radiation resistance strategies in two bacterial strains from the high background radiation area of Chavara-Neendakara: A comprehensive whole genome analysis
Source: PLoS One. 2024 Jun 10;19(6):e0304810. doi: 10.1371/journal.pone.0304810 (PMC11164402; doi:10.1371/journal.pone.0304810)
Supplement: S2 Table — (DOCX) [file pone.0304810.s019.docx]

**S2 Table.** **Other repair, recombination and replication genes for candidate strains, *D. radiodurans* and *E. coli***

| **Gene Name** | **Description** | **VITHBRA001** | **VITHBRA024** | ***D. radiodurans* Common genes** | ***E. coli*** |
| --- | --- | --- | --- | --- | --- |
| *polA* | DNA polymerase I | 1 | 1 | 1 | 1 |
| *polB* | DNA polymerase II | - | - | - | 1 |
| *polC* | DNA polymerase III PolC-type | 1 | 1 | - | - |
| *dnaE* | DNA polymerase III subunit alpha | 1 | 1 | 1 | 1 |
| *dnaX* | DNA polymerase III subunit gamma/tau | 1 | 1 | 1 | 1 |
| *dnaQ* | DNA polymerase III subunit epsilon | - | - | 1 | 1 |
| *dnaN* | Beta sliding clamp | 1 | 1 | 1 | 1 |
| *holA* | DNA polymerase III delta subunit | 1 | 1 | 1 | 1 |
| *holB* | DNA polymerase III delta prime subunit | 1 | 1 | - | 1 |
| *holC* | DNA polymerase III chi subunit | - | - | - | 1 |
| *holD* | DNA polymerase III psi subunit | - | - | - | 1 |
| *holE* | DNA polymerase III theta subunit | - | - | - | 1 |
| *polX* | DNA polymerase/3'-5' exonuclease PolX | 1 | 1 | 1 | - |
| *ligA* | DNA ligase | 1 | 1 | 1 | 1 |
| *ligB* | DNA ligase | - | - | 1(*DdrP*) | 1 |
| *topA* | DNA topoisomerase I | 1 | 1 | 1 | 1 |
| *top1B* | DNA topoisomerase IB | - | - | 1 | - |
| *topB* | DNA topoisomerase III | 2 | 1 | - | 1 |
| *pnp* | Polyribonucleotide nucleotidyltransferase | 1 | 1 | 1 | 1 |
| *xseA* | Exodeoxyribonuclease 7 large subunit | 1 | 1 | 1 | 1 |
| *xseB* | Exodeoxyribonuclease 7 small subunit | 1 | 1 | 1 | 1 |
| *sbcB* | Exonuclease I | - | - | - | 1 |
| *sbcC* | Exonuclease SbcC | 1 | 1 | 1 | 1 |
| *sbcD* | Exonuclease SbcD | 1 | 1 | 1 | 1 |
| *adaA* | Bifunctional transcriptional activator/DNA repair enzyme AdaA | 1 | 1 | - | 1 |
| *dnaC_1* | Replicative DNA helicase | 1 | - | - | - |
| *dnaC_2* | Replicative DNA helicase | 1 | 1 | DR_0549 | 1 |
| *dnaC_3* | Replicative DNA helicase | 1 | - | - | - |
| *dnaG* | DNA primase | 1 | 1 | 1 | 1 |
| *dnaB* | Replication initiation and membrane attachment protein | 1 | 1 | - | 1 |
| *dnaI* | Primosomal protein DnaI | 1 | 1 | - | - |
| *dnaA_1* | Chromosomal replication initiator protein DnaA | 1 | - | - | - |
| *dnaA_2* | Chromosomal replication initiator protein DnaA | 1 | 1 | 1 | 1 |
| *dnaD* | DNA replication protein DnaD | 1 | 1 | - | - |
| *dnaT* | DNA replication protein DnaT | - | - | - | 1 |
| *priA* | primosomal protein N' | 1 | 1 | 1 | 1 |
| *priB* | primosomal replication protein N | - | - | - | 1 |
| *priC* | primosomal replication protein N'' | - | - | - | 1 |
| *helD* | DNA helicase IV | 1 | 1 | 1 | 1 |
| *gyrB* | DNA gyrase subunit B | 1 | 1 | 1 | 1 |
| *gyrA* | DNA gyrase subunit A | 1 | 1 | 1 | 1 |
| *yoaA* | putative ATP-dependent DNA helicase YoaA | 1 | 1 | - | - |
| *yjcD* | Putative ATP-dependent DNA helicase YjcD | 1 | 1 | - | - |
| *recE* | exodeoxyribonuclease VIII | - | - | - | 1 |
| *umuC* | DNA polymerase V | - | - | - | 1 |
| *umuD* | DNA polymerase V | - | - | - | 1 |
| *dinB_1* | DNA polymerase IV | 1 | 1 | - | 1 |
| *dinB_2* | Putative UV-damage repair protein UvrX | 1 | 1 | - | - |
| *dcm* | Cytosine 5 methyltransferase | - | - | - | 1 |
