# Supplementary material for: Unraveling radiation resistance strategies in two bacterial strains from the high background radiation area of Chavara-Neendakara: A comprehensive whole genome analysis
Source: PLoS One. 2024 Jun 10;19(6):e0304810. doi: 10.1371/journal.pone.0304810 (PMC11164402; doi:10.1371/journal.pone.0304810)
Supplement: Attachment — Submitted filename: Response to Reviewers.docx [file pone.0304810.s020.docx]

**REBUTTAL LETTER**

**Comments of Reviewer 1:**

1. Introduction: (a) Remove the first paragraph that provides a short summary of the study, which is not needed.

**Ans:** The first paragraph is removed as suggested.

(b) page 3 line 62, ‘shown’ should be ‘known’.

**Ans:** ‘shown’ changed to ‘known’.

(c) page 3 line 64: DNA lesions are not caused by damaging nucleotide bases... this sentence needs to be rephrased.

**Ans:** The sentence has been rephrased as “Chromosomal DNA lesion such as DNA single strand breaks (SSB), double strand breaks (DSB) and damaged bases are considered to be hazardous effects of irradiation [1]”

(d) page 4: it would be helpful to provide a clear description of the factors that have been identified in earlier work as being key determinants of radiation resistance and I would suggest referring to figure 6 at this point in the introduction (and therefore moving Fig. 6 to Fig. 1).

**Ans:** (1) Earlier works mentioning the key determinants have been incorporated into the text (Introduction).

(2) With respect to the suggestion of the Reviewer1 for moving Fig 6 to Fig 1, we request that we may please be allowed to retain the position of Fig 6 in the result section as the figure was built with the intension of highlighting the main results of our study. Hence, we would request the Reviewer 1 to reconsider this suggestion. It may also please be noted that, as per the suggested clubbing of pictures 2 and 3, as well as 4 and 5, Fig 6 will now be numbered as Fig 4.

(e) page 4 lines 94-97: this sentence is quite unclear. The authors should expand this sentence to better explain what they mean and the role of these different pathways for non-experts. Also explain what the differences are, in which organisms etc.

**Ans:** Lines 94-97 has been elaborated to explain the roles of the pathways mentioned as well as their association with various organisms, as follows:

“*D. radiodurans*, for instance, relies on *ddrA, ddrB, ddrO* and *irrE (*also known as *pprI)* genes which are exclusive to them for efficient DNA repair [2,3]. Further, majority of the members of the genus *Deinococcus* (*D. radiodurans*, *D. geothermalis*, *D. deserti* and *D. murrayi*) and certain others such as *Rubrobacter radiotolerans* and *Micrococcus luteus* [4] depend highly onRecFOR system for recombinational repair of DNA while others such as *Keinococcus radiotolerans* [5], *Rhodococcus erythropolis* [6] and *Promicromonospora panici* [7] show the presence of both RecFOR and RecBCD pathways. Yet another variety, *Methylobacterium radiodurans*, depends only on RecBCD pathway [8] for repair mechanisms. For optimized maintenance of Mn/Fe ratio, which is crucial for ROS removal to ensure resistance, radio-resistant bacteria such as *D. radiodurans*, *D. geothermalis* [9] and *P. panici* [7] possess both MntABCD and NRAMP manganese transporter system. *R. radiotolerans* [4] *D. peraridilitoris* and *D. puniceus* [9], on the other hand, rely only on MntABCD transporter system”.

2. The word ‘discrepancies’ is used several times in this manuscript, but I think the authors mean ‘differences’. This should be corrected because the two words do not have the same meaning.

**Ans:** The word ‘discrepancies’ has been replaced with ‘differences’ in several places taking into account the propriety of the usage.

3. Acronyms should be explained and spelled out when first used.

**Ans:** Acronyms have been explained and spelled out when first used.

4. Methods: what dose rate was used for the irradiation? How long did the irradiation last?

**Ans:** Irradiation was conducted at IGCAR, Kalpakkam (a centre under the department of Atomic Energy, government of India) using the cobalt-60 facility with dose rate of 0.77 kGy/hr. Dosage of 1 kGy will be attained in ~1 hr 18 min, while 2, 3, 4, 5 kGy will be attained in ~2 hr 36 min, ~3hr 54 min, ~5 hr 12 min and ~6 hr 30 min respectively. This information is incorporated in the manuscript in the “materials and methods” section.

5. Results – Survival curves: The survival plots look somewhat strange. What is plotted? Are these the mean values of the triplicate measurements? The plot for VITHBRA024 is clearly not a linear regression; it actually seems to reach a plateau after a sudden drop. It is actually surprising to see a linear regression for the other strain, which is quite unusual. It would be good to have data for lower doses also.

**Ans:** (1) Survival graph (Fig. 1) is plotted using the mean values of the triplicate measurements. Results of previous investigations demonstrate that the D_10_ plots could be linear or non-linear, depending on the species in question [10,11]; similar pattern was observed in our D_10_ graph for VITHBRA001 and VITHBRA024. In the manuscript, D_10_ was calculated through linear regression analysis adopted from Kortei et al., (2017) [10]. Sample values were re-examined and repeated to ensure accuracy. In this scenario, we are encouraged to justify both the D_10_ graphs; the difference in the course of diminution could be due to the difference in the rate of bacterial death against increasing dose of irradiation.

(2) As the present study was aimed at performing WGS of radiation resistant bacteria (D_10_ >1 kGy), data for doses lower than 1 kGy were not taken, hence, not available.

6. Results – Fig. 2 & 3: figures 2 and 3 could be combined into a single figure with two panels. It would be interesting to know how distantly related the two strains are. Would it be possible to show a phylogenomic tree with both strains visible in addition to the more focused trees on each of the individual strains?

**Ans:** (1) In the revised manuscript, the two figures (Fig 2 and Fig 3) have been combined into a single figure as suggested, and labelled as Fig 2; figure legends are modified accordingly.

(2) Phylogenomics tree has been constructed through online TYGS webserver, using the closely allied strains mentioned in the more focussed trees (Fig 2), with *D. radiodurans,* been treated as an outgroup. The tree shows both the candidate strains in two separate clades. This is being included as supplement data (S1 Fig).

7. Results – Genome annotation: The authors used different annotation programs to analyse their assembled genomes. As a non-expert, which of these annotations can we trust? Can the authors briefly describe the differences? What was used for the rest of the study?

**Ans:** Genome annotation was carried out using three annotators such as Prokka, RAST and PGAP. All the three annotators are valid and trustworthy, and provide high quality output. However, due to the differences in the reference database used, and the computational speed of annotation, with a given annotator, some of the distant genes are marked “hypothetical” [12]. For example: the gene *recJ* was identified as “hypothetical gene” by Prokka annotator for both the organisms VITHBRA001 and VITHBRA024; but it was annotated as *recJ* by both RAST and PGAP. Another example is with gene *nudF* which was annotated as “Nudix hydrolase” by PGAP for both VITHBRA001 and VITHBRA024, without specifying the exact function of the gene. Comparison with Prokka and RAST, however, has helped us identify the function of the gene as ADP-ribose pyrophosphatase. These reasons prompted us to use a combination of annotators for the present study. This has been mentioned in the “Results” section of the revised manuscript. For all the further analyses such as COG, KEGG, CD search, standalone BLAST and Uniprot, we have used the sequences generated by Prokka annotator as it was efficient and convenient to use. This has been incorporated into the manuscript under “Materials and Methods” section.

8. Results – Gene classification: As for figures 2 and 3, I suggest combining figures 4 and 5 into a single figure with 2 panels. It would be interesting and helpful for the comparison to include a radiation resistant (eg . *Deinococcus radiodurans*) and a radiation sensitive (eg. *Escherchia coli*) bacteria in these plots for comparison. To reliably compare the number of genes under different categories, the authors should use statistical tests and report on these to determine whether the observed differences are statistically significant or not.

**Ans:** (1) Figures 4 and 5 have been merged as figure 3 and the figure legends have been modified accordingly.

(2) As suggested by the Reviewer, along with Figure 3, the COG data from *D. radiodurans* and *E. coli* have been incorporated (please see the figure attached). However, admittedly, the expected proportionate difference in the distribution of coding sequences (gene copies), as evolved from COG analysis, does not match with the candidates’ already established resistance capability. Apparently, this (unexplainable) discrepancy could be due to the software’s inherent limitation in specifically classifying/identifying the genes. Further, significantly, the comparison of the COG data presented in the paper has been made between closely allied species (*Metabacillus* and *Bacillus*), almost belonging to the same genus [*Metabacillus* was considered under the genus *Bacillus* until 2020]. Furthermore, in one of the previous studies using COG database, the authors (Ogwu et al. 2019) [13], have successfully made a comparative genomic analysis involving five species from the genus *Deinococcus* and Liu et al., (2022) [14] has done the same using closely allied *Sphingomonas* species. It looks as though the species with orthological/phylogenetic closeness would be suitable for successful COG data analysis. Further, Galperin et al., (2017) has mentioned the use of COG for analysis of protein families in the “genomic neighbourhood” [15]. We would seek the advice from the Reviewer in this regard, with respect to the propriety of inclusion of data from *D. radiodurans* and *E. coli* in Figure 3.

(3) As the data do not show any variance in spite of repeated analysis, and standard deviation thus being zero, we are unable to find an appropriate statistical analysis for comparing the values of COG and KEGG data. We would gratefully appreciate suggestions if any, from the Reviewer in this regard.

9. Results – Genes involved in radiation resistance: (a) Remove first paragraph (lines 319-324 page 15), which is a repeat of the introduction, and simply introduce the analysis with a short sentence explaining the rationale for the analysis of selected radiation resistance factors.

**Ans:** The first paragraph is removed as suggested. Rationale for the analysis has been mentioned in the succeeding paragraph.

(b) What about the SOS response factors? The authors mention the alternative system found in *Deinococcus* (the RDRM system) and note that it is missing in their strains. So, do these two strains have a classical SOS response system? Also, there is no mention of the photolyase, an important enzyme for UV-induced DNA damage which is missing in *D. radiodurans*.

**Ans:** (1) We have added a section explaining the presence of SOS response in candidate strains under the heading “DNA damage response (DDR)”.

(2) We have mainly focused on the possible strategies that help in ionizing radiation resistance hence we did not scrutinize the presence of photolyase which is essential for UV radiation resistance.

(c) Importantly, this whole section would benefit from including *D. radiodurans* (as an example of a radiation resistant bacterium) and *E. coli* (as an example of a radiation sensitive bacterium) in the various tables presenting the genes potentially involved in radiation resistance (Tables 2 to 12). This would make it easier to compare and to highlight the genes that are associated with radiation resistance, those which are found in all bacteria and those which are missing in radiation resistant bacteria (eg RecBCD). Table 13 could then focus more particularly on the similarities and differences with *D. radiodurans*.

**Ans:** Tables 2-12 have been incorporated with the results from *E. coli*, so as to have representations from radiation resistant (*D. radiodurans*) and radiation sensitive (*E. coli*) bacteria. The difference in genetic constitution and its possible impact are discussed under “Comparison of the candidate strains with *D. radiodurans* and *E. coli* – a consolidated genomic approach”

(d) As it is written, it sounds like the presence of DNA repair genes is associated with radiation resistance, but this is incorrect and should be clarified. Most of the genes involved in DNA repair are present in all bacteria and are typically highly conserved. The authors should thus focus on the differences, eg. expanded families (UvrA2, extra UvrC, AlkA), high number of DNA glycosylases and Nudix hydrolases etc.

**Ans:** (1) Phenomena such as ROS removal and protein protection are important ways to tackle radiation resistance. And we do admit that DNA repair is not the exclusive mechanism associated with radiation resistance. The manuscript has been revised with examples of methods other than DNA repair to be associated with radiation resistance.

(2) The importance of expanded families UvrA2 has been highlighted with help of figures that were initially provided in the supplement but now shifted in the text as main figure. Possible function and structure of UvrC was already discussed in the NER section of manuscript. Possible function of AlkA was suggested in BER section. *E. coli* also seems to have high number of glycosylases, but multiple number of copies of these genes present in *D. radiodurans* and VITHBRA001 could be contributing substantially to the high radiation resistance. Further, nudix hydrolases have been discussed appropriately in the Nudix dedicated section as well as in the section entitled “comparison of the candidate strains, *D. radiodurans* and *E. coli*- a consolidated genomic approach”.

10. Conclusions: this could be shortened; the manuscript is already very long.

**Ans:** The conclusion has been shortened by shifting some of the points to the “introduction” and “evolutionary perspective”.

**Comments of Reviewer 2:**

1. The introduction lacks detailed discussion on studying mid-range radiation-resistant bacteria's significance, especially regarding biotechnological applications and environmental bioremediation. Elaborate on contextual importance and key knowledge gaps addressed.

**Ans:** We have now addressed the significance of the studies on mid-range radiation resistant bacteria in “Introduction” with respect to environmental bioremediation of radionuclides and its biotechnological application (lines 87-105). The ‘Introduction’ also addresses the contextual importance and the knowledge gaps existing in this regard.

2. Please include more information on statistical methods used for data analysis, including specific tests, confidence intervals, and significance levels related to survival curves, D10 values, and comparative genomics. This would strengthen reliability of conclusions drawn.

**Ans:** Statistical analysis of the survival graphs has been included in the figure mentioning the confidence intervals, significance level and R squared values. The mean values which were used to plot the graph have been checked for normal distribution using Shapiro-Wilk normality test, and the data suggests that at 95% significance level, the raw data are normally distributed. Additionally, R^2^ value for both the graphs suggest that X-axis and Y-axis are strongly correlated thus the survival of the organisms are dose-dependent. We could also determine that the regression line is significantly non-zero with a confidence of > 99% suggesting that the D_10_ values of both the strains are significantly different, further reiterating the difference in radiation tolerance existing between the two strains.

3. The section on comparison with *Deinococcus radiodurans* is useful. Please expand comparison analysis to include additional radiation-resistant and susceptible strains for broader evolutionary context elucidating genetic basis across bacterial lineages.

**Ans:** In the manuscript we have incorporated information of other radiation resistant bacteria such as *Rubrobacter radiotolerans*, *Keinococcus radiotelerans*, *Hymenobacter* species, other *Deinococcus* species and *Priestia megaterium,* in addition to that of *Deinococcus radiodurans*. Under the “Result and discussion” section we will see the mention of these bacteria under homologous recombination, base excision repair, nucleotide excision repair, catalases, SODs, thioredoxin system, Mn/Fe homeostasis, and comparison between candidate strains-a genomic approach. Additionally, we have now incorporated radiation sensitive bacteria *E. coli* for a better comparison as suggest by the Reviewers along with the comparative gene tables 2-12 and an additional supplement table S2_Table.

4. There is a lot of detail in the results section, and it makes it hard for the readers to comprehend the main points. Please consider moving some supplemental data to figures or tables and highlighting only the most significant findings in the main text.

**Ans:** As per Reviewer’s suggestion, in order to highlight the significant findings, 11 supplement figures have been moved to the main text. Accordingly, supplements S7 and S8 are merged as Fig 5, S9 as Fig 6, S10 and S11 merged as Fig 7, S12 as Fig 8, S21-S24 as Fig 9 to Fig 12 and S26 as Fig 13. Further, the remaining supplements were re-numbered.

5. Please ensure high quality, clear resolution for all figures and graphs. Carefully review and revise language for clarity and conciseness.

**Ans:** We have constructed figures based on PLoS ONE requirements with 300 dpi resolution. We used the PACE software provided by PLoS ONE to check the figure requirements for PLoS ONE.

6. Experimental validation via gene knockouts or overexpression would significantly reinforce inferred roles of genes/pathways in radiation resistance.

**Ans:** Comments from the Reviewer is fully accepted. Wherever possible, results of the present study have been compared with the those of gene knockouts and/or expression studies carried out in established experimental models such as *D. radiodurans* and *E. coli*, and incorporated under “Results and discussion”. Obviously, future attempts involving expression studies would give conclusive information on the specific role of the genes involved in radiation resistance.

7. Expand discussion of findings’ ecological and evolutionary implications, including potential evolution mechanisms of identified genes/pathways.

**Ans:** Answer 7 and 9 are clubbed under the heading “Evolutionary perspective”. The first paragraph portrays the eco- evolutionary perspective of radiation resistance and the text is as follows:

“The evolution of radio-resistance has been a subject of discussion for decades. From eco-evolutionary perspective, radio-resistance is thought to be an aftermath of resistance to several common stressors [16,17]. Radiation-resistant extremophiles are scattered and have been isolated from diverse environments [18,19], which indicates that the radiation-resistant extremophiles do not have a common ancestor. Deserts have been observed to be the most common habitat to find radiation-resistant extremophiles [20–22]. There are suggestions on ROS-scavenging system as the interlinkage that enables the microbes to resist many different stresses including radiation [23]. There exists a general argument that radiation resistance has been attained through desiccation [24]. Apparently, this is applicable for most of the highly resistant varieties (including *Deinococcus sp.*). However, that desiccation as a stressor is applicable in the resistance of the candidate strains, is enigmatic, as these species have been exposed to low-dose radiation from time immemorial (also Bruckbauer and Cox, 2021) [24]. Earlier, as a first-time study, our laboratory had isolated 35 bacterial species from this HBRA soil [25]. Out of these, 32, selected at random, were subjected to gamma irradiation to the tune of 1-5 kGy. Interestingly, eight of them had D10 value of >1 kGy and survived 5 kGy, indicating that a sizeable percentage (25%) of the bacterial population of this HBRA could be considered as IRRB. It is tempting to suggest that this sizeable proportion of radiation resistance, shown by the microbial population, could be the (hormetic) aftermath of long-term exposure to low-dose radiation prevalent in the HBRA”.

8. Expand discussion of findings’ ecological and evolutionary implications, including potential evolution mechanisms of identified genes/pathways. Address limitations in relying solely on D10 values as radiation resistance measure, depth of analysis, unidentified genes. Discuss future directions addressing these gaps.

**Ans:** Notwithstanding the wide use of D10 values for estimating the radiation sensitivity, it also deserves to mention the limitations of the method, i.e., inaccurate interpretation of dose measurements (caused by exposure of the sample, resuspension medium, available oxygen, and dose uniformity ratio) can result in inaccurate D10 –values, raising a sense of caution to avoid any potential experimental errors. Such inaccuracies could lead to over- and under-estimation of the irradiation treatment. However, in our experiments, the accurate dosage measurement was ensured at IGCAR, where the sample was irradiated taking special care to avoid any kind of experimental error.

**Unidentified genes:** A comprehensive analysis of COG data of our candidate strains shows that almost 3.92% of the proteome has unidentified function as categorized under COG with an additional 24.19% of proteome not characterized by COG. Thus, total 28.11% of the VITHBRA001 proteome is unidentified. Further, VITHBRA024 have 4.02% of the proteome under “unidentified function” category of COG with an additional 22.10% of proteome not characterized by COG. This makes a total of 26.12% of the VITHBRA024 genome having unidentified genes. That the unknown function aspect of candidate genomes could hold more knowledge of their radiation resistance property has been suggested in manuscript from lines 351-353. The unidentified genes of radiation resistance could be further established through transcriptomics studies and such approaches were also taken when studying the radiation resistant bacterium *D. radiodurans*. This information in incorporated into the manuscript under the section “Annotation of assembled genome”.

Limitation to depth of analysis is mentioned in the “conclusion” section of manuscript.

9. The discussion of functional redundancy and pleiotropy of genes is lacking, making it difficult to definitively assign specific roles in radiation resistance.

**Ans:** Answers of query 7 and 9 are clubbed together under the heading “Evolutionary perspective”. The 2^nd^ and 3^rd^ paragraph mentions about redundancy and pleiotropy respectively. The text is as follows:

“In both the candidate species, we could observe instances of gene redundancy as well as functional redundancy that invites our attention, a matter of evolutionary significance. Gene level redundancy has been suggested to result in increased genetic robustness as it may 'protect' the organism in question from potentially harmful mutations, and help better environmental adaptation [26–28]. It has already been proven that redundancies among genes involved in reactive oxygen species (ROS) clearance can promote radio-resistance of *D. radiodurans*, inasmuch as deficient genes (mutant ones for example) could be substituted by a group of normal genes which are kept in reserve to act as soon as the situation demands [29]. One of the examples to be cited from the candidate specimen (VITHBRA001) for gene redundancy is UvrA (A1 and A2), involved in DNA repair; UvrA2 could also be involved in repair in the absence of UvrA1 [30]. Further, MnKat and catalases, involved in removal of reactive oxygen, and AddAB and RecQ RecJ, involved in DNA end-resection could also be listed under functional redundancy in the candidate species.

We also examined the possible role of pleiotropic genes, if any, in the candidate species since such genes are known to contribute to radiation resistance. A pleiotropic protein (PprA) from *D. radiodurans* has been found to substantially help in radiation resistance through DSB repair. Also, this gene when transfected in *E. coli* shows increased catalase activity assisting higher tolerance to H_2_O_2_ [31,32]. Another pleotropic protein RecQ, with its three tandem HRDC domains, known to regulate DNA replication, recombination repair, nonhomologous end-joining, telomere maintenance, and damage response has been demonstrated to involve in radiation resistance [33]. Significantly, such pleiotropic genes are not ubiquitous, but thus far reported only from *D. radiodurans*. However, in the present study, *pnpA* gene, known for its pleiotropic function has been observed in both the candidate strains. It has 3’-5’ exoribonuclease activity to remove damaged ribonucleotides [34], basal trimming activity at the DSBs of DNA for HR and NHEJ pathways [35,36]. DR is reported to have *pnp* gene, important in removing 8-oxo-ribonucleotide, formed after IR exposure and may also interact with other DNA repair genes [37]”.

**References**

1.	Ward JF. Biochemistry of DNA lesions. Radiat Res Suppl. 1985;8: S103-111.

2.	Jung KW, Lim S, Bahn YS. Microbial radiation-resistance mechanisms. Journal of Microbiology. 2017;55: 499–507. doi:10.1007/s12275-017-7242-5

3.	Daly MJ. The scientific revolution that unraveled the astonishing DNA repair capacity of the *Deinococcaceae* : 40 years on. Can J Microbiol. 2023;69: 369–386. doi:10.1139/cjm-2023-0059

4.	Egas C, Barroso C, Froufe HJC, Pacheco J, Albuquerque L, da Costa MS. Complete genome sequence of the radiation-resistant bacterium Rubrobacter radiotolerans RSPS-4. Standards in Genomic Sciences. 2015;9: 1062–1075. doi:10.4056/sigs.5661021

5.	Bagwell CE, Bhat S, Hawkins GM, Smith BW, Biswas T, Hoover TR, et al. Survival in nuclear waste, extreme resistance, and potential applications gleaned from the genome sequence of Kineococcus radiotolerans SRS30216. PLoS ONE. 2008;3. doi:10.1371/journal.pone.0003878

6.	Gouvêa Taketani R, Domingues Zucchi T, Soares de Melo I, Mendes R. Whole-Genome Shotgun Sequencing of Rhodococcus erythropolis Strain P27, a Highly Radiation-Resistant Actinomycete from Antarctica. Genome Announcements. 2013;1: 10.1128/genomea.00763-13. doi:10.1128/genomea.00763-13

7.	Guesmi S, Nouioui I, Pujic P, Dubost A, Najjari A, Ghedira K, et al. Draft genome sequence of Promicromonospora panici sp. nov., a novel ionizing-radiation-resistant actinobacterium isolated from roots of the desert plant Panicum turgidum. Extremophiles. 2021;25: 25–38. doi:10.1007/s00792-020-01207-8

8.	Maeng S, Kim D-U, Lim S, Lee B-H, Lee K-E, Kim M, et al. Methylobacterium radiodurans sp. nov., a novel radiation-resistant Methylobacterium. Arch Microbiol. 2021;203: 3435–3442. doi:10.1007/s00203-021-02293-8

9.	Lim S, Jung J-H, Blanchard L, de Groot A. Conservation and diversity of radiation and oxidative stress resistance mechanisms in Deinococcus species. FEMS Microbiology Reviews. 2019;43: 19–52. doi:10.1093/femsre/fuy037

10.	Kortei NK, Odamtten GT, Obodai M, Wiafe‐Kwagyan M. Mycofloral profile and the radiation sensitivity (D10 values) of solar dried and gamma irradiated Pleurotus ostreatus (Jacq.Ex. Fr.) Kummer fruitbodies stored in two different packaging materials. Food Sci Nutr. 2017;6: 180–188. doi:10.1002/fsn3.545

11.	Porfiri L, Burtscher J, Kangethe RT, Verhovsek D, Cattoli G, Domig KJ, et al. Irradiated Non-replicative Lactic Acid Bacteria Preserve Metabolic Activity While Exhibiting Diverse Immune Modulation. Front Vet Sci. 2022;9. doi:10.3389/fvets.2022.859124

12.	Ruiz-Perez CA, Conrad RE, Konstantinidis KT. MicrobeAnnotator: a user-friendly, comprehensive functional annotation pipeline for microbial genomes. BMC Bioinformatics. 2021;22: 11. doi:10.1186/s12859-020-03940-5

13.	Ogwu MC, Srinivasan S, Dong K, Ramasamy D, Waldman B, Adams JM. Community Ecology of Deinococcus in Irradiated Soil. Microbial Ecology. 2019;78: 855–872. doi:10.1007/s00248-019-01343-5

14.	Liu Y, Cui X, Yang R, Zhang Y, Xu Y, Liu G, et al. Genomic Insights into the Radiation-Resistant Capability of Sphingomonas qomolangmaensis S5-59T and Sphingomonas glaciei S8-45T, Two Novel Bacteria from the North Slope of Mount Everest. Microorganisms. 2022;10: 2037. doi:10.3390/microorganisms10102037

15.	Galperin MY, Kristensen DM, Makarova KS, Wolf YI, Koonin EV. Microbial genome analysis: the COG approach. Briefings in Bioinformatics. 2019;20: 1063–1070. doi:10.1093/bib/bbx117

16.	Billi D, Wright DJ, Helm RF, Prickett T, Potts M, Crowe JH. Engineering Desiccation Tolerance inEscherichia coli. Applied and Environmental Microbiology. 2000;66: 1680–1684. doi:10.1128/AEM.66.4.1680-1684.2000

17.	Bauermeister A, Moeller R, Reitz G, Sommer S, Rettberg P. Effect of Relative Humidity on Deinococcus radiodurans’ Resistance to Prolonged Desiccation, Heat, Ionizing, Germicidal, and Environmentally Relevant UV Radiation. Microb Ecol. 2011;61: 715–722. doi:10.1007/s00248-010-9785-4

18.	Gérard J-C, Gustin J, Grodent D, Delamere P, Clarke JT. Excitation of the FUV Io tail on Jupiter: Characterization of the electron precipitation. Journal of Geophysical Research: Space Physics. 2002;107: SMP 30-1-SMP 30-7. doi:10.1029/2002JA009410

19.	Yu LZH, Luo XS, Liu M, Huang Q. Diversity of ionizing radiation-resistant bacteria obtained from the Taklimakan Desert. Journal of Basic Microbiology. 2015;55: 135–140. doi:10.1002/jobm.201300390

20.	Paulino-Lima IG, Azua-Bustos A, Vicuña R, González-Silva C, Salas L, Teixeira L, et al. Isolation of UVC-Tolerant Bacteria from the Hyperarid Atacama Desert, Chile. Microb Ecol. 2013;65: 325–335. doi:10.1007/s00248-012-0121-z

21.	Paulino-Lima IG, Fujishima K, Navarrete JU, Galante D, Rodrigues F, Azua-Bustos A, et al. Extremely high UV-C radiation resistant microorganisms from desert environments with different manganese concentrations. Journal of Photochemistry and Photobiology B: Biology. 2016;163: 327–336. doi:10.1016/j.jphotobiol.2016.08.017

22.	Hezbri K, Ghodhbane-Gtari F, Montero-Calasanz M del C, Nouioui I, Rohde M, Spröer C, et al. Geodermatophilus pulveris sp. nov., a gamma-radiation-resistant actinobacterium isolated from the Sahara desert. International Journal of Systematic and Evolutionary Microbiology. 2016;66: 3828–3834. doi:10.1099/ijsem.0.001272

23.	Imlay JA. The molecular mechanisms and physiological consequences of oxidative stress: lessons from a model bacterium. Nat Rev Microbiol. 2013;11: 443–454. doi:10.1038/nrmicro3032

24.	Bruckbauer ST, Cox MM. Experimental evolution of extremophile resistance to ionizing radiation. Trends in Genetics. 2021;37: 830–845. doi:10.1016/j.tig.2021.04.011

25.	Sowptika P, Anilkumar G. The bacterial population inhabiting a top-ranking High Background Radiation Area: A first-time report. Journal of Environmental Biology. 2023;44: 45–56. doi:10.22438/jeb/44/1/MRN-5018

26.	Patra B, Kon Y, Yadav G, Sevold AW, Frumkin JP, Vallabhajosyula RR, et al. A genome wide dosage suppressor network reveals genomic robustness. Nucleic Acids Res. 2017;45: 255–270. doi:10.1093/nar/gkw1148

27.	Sanchez-Perez G, Mira A, Nyirő G, Pašić L, Rodriguez-Valera F. Adapting to environmental changes using specialized paralogs. Trends in Genetics. 2008;24: 154–158. doi:10.1016/j.tig.2008.01.002

28.	Wagner A. Genetic redundancy caused by gene duplications and its evolution in networks of transcriptional regulators. Biol Cybern. 1996;74: 557–567. doi:10.1007/BF00209427

29.	Liu F, Li N, Zhang Y. The radioresistant and survival mechanisms of Deinococcus radiodurans. Radiation Medicine and Protection. 2023;4: 70–79. doi:10.1016/j.radmp.2023.03.001

30.	Tark M, Tover A, Koorits L, Tegova R, Kivisaar M. Dual role of NER in mutagenesis in *Pseudomonas putida*. DNA Repair. 2008;7: 20–30. doi:10.1016/j.dnarep.2007.07.008

31.	Kota S, Misra HS. PprA: A protein implicated in radioresistance of Deinococcus radiodurans stimulates catalase activity in Escherichia coli. Appl Microbiol Biotechnol. 2006;72: 790–796. doi:10.1007/s00253-006-0340-7

32.	Kota S, Charaka VK, Misra HS. PprA, a pleiotropic protein for radioresistance, works through DNA gyrase and shows cellular dynamics during postirradiation recovery in Deinococcus radiodurans. J Genet. 2014;93: 349–354. doi:10.1007/s12041-014-0382-z

33.	Chen H, Huang L, Hua X, Yin L, Hu Y, Wang C, et al. Pleiotropic effects of RecQ in Deinococcus radiodurans. Genomics. 2009;94: 333–340. doi:10.1016/j.ygeno.2009.08.001

34.	Cameron TA, Matz LM, Lay NRD. Polynucleotide phosphorylase: Not merely an RNase but a pivotal post-transcriptional regulator. PLOS Genetics. 2018;14: e1007654. doi:10.1371/journal.pgen.1007654

35.	Cardenas PP, Carrasco B, Sanchez H, Deikus G, Bechhofer DH, Alonso JC. Bacillus subtilis polynucleotide phosphorylase 3′-to-5′ DNase activity is involved in DNA repair. Nucleic Acids Research. 2009;37: 4157–4169. doi:10.1093/nar/gkp314

36.	Cardenas PP, Carzaniga T, Zangrossi S, Briani F, Garcia-Tirado E, Dehò G, et al. Polynucleotide phosphorylase exonuclease and polymerase activities on single-stranded DNA ends are modulated by RecN, SsbA and RecA proteins. Nucleic Acids Research. 2011. doi:10.1093/nar/gkr635

37.	Han R, Jiang J, Fang J, Contreras LM. PNPase and RhlB Interact and Reduce the Cellular Availability of Oxidized RNA in Deinococcus radiodurans. Microbiology Spectrum. 2022;10: e02140-22. doi:10.1128/spectrum.02140-22
