# Supplementary material for: Unraveling radiation resistance strategies in two bacterial strains from the high background radiation area of Chavara-Neendakara: A comprehensive whole genome analysis
Source: PLoS One. 2024 Jun 10;19(6):e0304810. doi: 10.1371/journal.pone.0304810 (PMC11164402; doi:10.1371/journal.pone.0304810)
Supplement: Attachment — Submitted filename: Response to Reviewers.docx [file pone.0304810.s021.docx]

**Rebuttal Letter**

**Editor’s comments:**

***Query 1.*** *Attention is invited for rebuttal in response to Reviewer 1's comment 1e. How RecBCD presence or absence can influence recombination repair is well taken with examples. Does RecBC really has a role in DSB repair (believed in some bacteria) was addressed in D. radiodurans using E coli RecBC. A mentioned on this aspect may help to advance our understanding on the paradigm that RecBC(D) as a recombination repair enzyme.*

**Ans:** The possible role of RecBC in DSB repair has been discussed in the text, in light of the expression of *E. coli recBC* in recombinant *D. radiodurans* (Khairnar *et al.,* 2007); this has been incorporated into the text under the Section “Homologous recombination (HR)”. Line no. 386-391 of Page no. 18 in “Manuscript”.

***Query 2.*** *Rev.1, comment 4. Dose rate in this work is found to be moderate (.77kGy/h). Data compared from others are mostly high dose rate. While some work has also been done in D. radiodurans with low dose rate. Authors should try to discuss dose rate factor with appropriate citation.*

**Ans:** This moderate dose rate (0.77 kGy/hr) used for the present irradiation experiment, proves to be ideal for estimating the impact of IR on bacterial cells as it offers a smooth graph depicting a discernible decline in the bacterial population against increased radiation dosage, allowing a clear estimate of D_10_ values. Further, previous investigators have used dose rates coming in close proximity (0.7 to 0.8 kGy/hr) to what was used in the present study (eg: *Staphylococcus aureus*, dose rate 0.78 kGy, Dion et al. 1994; *Bacillus subtilis* and *E. coli*, dose rate 0.7 kGy, Hing et al. 2022). We have incorporated this information in the “materials and method” section. Line no. 160-161 of Page no. 7 in “Manuscript”.

***Query 3.*** *Some of the rebuttal components are not seen in revised tracked copy of the manuscript. May recheck it please.*

**Ans:** We have taken utmost care in addressing all the questions posed by the Reviewers and made the corresponding changes in the “Revised manuscript with track changes”.

Nevertheless, we are unsure if we have, by oversight, left any point unattended. May we seek the kind advice from the Reviewer/Editor in this regard.

***Query 4****. Although, PLOS ONE does not restrict to the size of the paper and references, the quality of referencing is advised. I could see some of the citations are redundant and can be removed. Please check the quality of work, journals and originality in the work that are being cited in manuscript. If possible, bring down number of citations to close to 150 from the current number more than 200.*

**Ans:** We have made sincere efforts in removing any redundancy in the references cited in the paper so as to reduce the number of references as much as possible. Accordingly, the number has been brought down from 208 to 194; further reduction, we are afraid, might lead to loss of information in the manuscript.

***Query 5****. I suggest checking the manuscript carefully for English language and composition.*

**Ans:** We have thoroughly checked the paper for English composition and made a few changes to further easy readability.
