# Supplementary material for: Unraveling radiation resistance strategies in two bacterial strains from the high background radiation area of Chavara-Neendakara: A comprehensive whole genome analysis
Source: PLoS One. 2024 Jun 10;19(6):e0304810. doi: 10.1371/journal.pone.0304810 (PMC11164402; doi:10.1371/journal.pone.0304810)
Supplement: Attachment — Submitted filename: Response to Reviewers.docx [file pone.0304810.s022.docx]

**Rebuttal Letter**

**Query 1:** Please check details and authenticity of information given in Table 13. Some exclusive genes in your isolates seems to be there in *Deinoocccus radiodurans* (e.g. UvsE, J Bacteriol. 2002 Feb; 184(4): 1003–1009).

**Ans:** The details and authenticity have been checked as per the comments from the Reviewer and the Table 13 has been revised. Accordingly, VITHBRA001 has two UvsE homologs as compared to one in *D. radiodurans*. The UvsE (VITHBRA001_01817) that is mentioned as ‘exclusive’ in Table 13, shares lesser identity with *D. radiodurans* UvsE (DR_1819), as compared to the other homolog of VITHBRA001 UvsE (VITHBRA001_00228). The latter is mentioned as the common gene/protein under the column “**VITHBRA001+ Dra”** of Table 13. Thus, to differentiate, we have now mentioned the locus tag of the exclusive gene/protein of VITHBRA001, and other exclusive genes/proteins as well, particularly for the ones which have another homolog and share the same name. Please see the Table 13, and the track changes for the same.
